# Abnormal Glucose Metabolism in Male Mice Offspring Conceived by *in vitro* Fertilization and Frozen-Thawed Embryo Transfer

**DOI:** 10.3389/fcell.2021.637781

**Published:** 2021-02-09

**Authors:** Ningxin Qin, Zhiyang Zhou, Wenlong Zhao, Kexin Zou, Weihui Shi, Chuanjin Yu, Xia Liu, Zehan Dong, Yiting Mao, Xinmei Liu, Jianzhong Sheng, Guolian Ding, Yanting Wu, Hefeng Huang

**Affiliations:** ^1^The International Peace Maternity and Child Health Hospital, School of Medicine, Shanghai Jiao Tong University, Shanghai, China; ^2^Shanghai Key Laboratory of Embryo Original Diseases, Shanghai Jiao Tong University, Shanghai, China; ^3^Hospital of Obstetrics and Gynecology, Fudan University, Shanghai, China; ^4^Department of Pathology and Pathophysiology, School of Medicine, Zhejiang University, Hangzhou, China; ^5^The Key Laboratory of Reproductive Genetics, Ministry of Education, School of Medicine, Zhejiang University, Hangzhou, China

**Keywords:** frozen-thawed embryo transfer, glucose metabolism, insulin resistance, PI3K/AKT pathway, transcriptome

## Abstract

Frozen and thawed embryo transfer (FET) is currently widely applied in routine assisted reproductive technology (ART) procedure. It is of great necessity to assess the safety of FET and investigate the long-term effect including glucose metabolism on FET-conceived offspring. The mouse model is a highly efficient method to figure out the relationship between the process of FET and offspring health. In this study, we obtained mouse offspring of natural conception (NC), *in vitro* fertilization (IVF), and FET. Glucose and insulin tolerance test (GTT/ITT) were performed on both chow fed or high fat diet (HFD) fed offspring to examine the glucose metabolism status. We detected hepatic PI3K/AKT pathway by western blotting and transcriptome status by RNA-sequencing. Impaired glucose tolerance (IGT) and decreased insulin tolerance were occurred in FET conceived male offspring. After challenged with the HFD-fed, male offspring in FET group performed earlier and severer IGT than IVF group. Furthermore, higher HOMA-IR index and higher serum insulin level post glucose injected in FET-chow group suggested the insulin resistance status. The PI3K/AKT signaling pathway, the major pathway of insulin in the liver, were also disrupted in FET group. Transcriptomics of the liver reveals significantly downregulated in glucose metabolic process and insulin resistance in the FET-chow group. In our study, FET-conceived male mouse offspring presented glucose metabolism dysfunction mainly manifesting insulin resistance. The hepatic insulin signaling pathway were in concordance with reduced glycogen synthesis, increased glycolysis and enhanced gluconeogenesis status in FET-conceived male offspring.

## Introduction

Since the first baby born from *in vitro* fertilization and embryo transfer (IVF-ET) in 1978, assisted reproductive technologies (ART) have rapidly developed over the past decades. More than 8 million newborns have been conceived by ART (European IVF monitoring Consortium et al., 2017). However, children conceived by ART are at risk of adverse short-term and long-term effects, such as neurodevelopmental disorders, poor school performance in childhood and cardiometabolic dysfunction, which has raised concerns by many researchers (Savage et al., 2011; Sandin et al., 2013; Sharpe, 2018; Gu et al., 2019; Hargreave et al., 2019; Pinborg, 2019; Cui et al., 2020). In 1983, frozen-thawed embryo transfer (FET) was first introduced by Trounson and Mohr (1983). Over the last decade, the use of FET cycles in ART programs has maintained an increasing trend worldwide (European IVF monitoring Consortium et al., 2017).

Compared with fresh embryo transfer, FET cycles appear to show better obstetric and fetal outcomes, such as lower risks of low birth weight, small for gestational age and extremely preterm birth (Sha et al., 2018; Gu et al., 2019; Roque et al., 2019). However, some researchers have reported potential disadvantages in FET neonatal outcomes, such as increased risks of pregnancy-induced hypertension, large for gestational age and macrosomia (Berntsen and Pinborg, 2018; Sha et al., 2018; Roque et al., 2019; Wei et al., 2019), which are important risk factors for obesity, cardiovascular complications and metabolic dysfunction (Boney et al., 2005; Hermann et al., 2010; Ornoy, 2011). Several follow-up studies have suggested that newborns conceived by FET often show abnormal lipid metabolism and an increased risk of childhood cancer compared with children born after natural conception and fresh embryo transfer (Green et al., 2013; Hargreave et al., 2019). However, the limited clinical applications duration of FET prevented researchers from investigating the long-term effects of FET on human offspring.

To eliminate the complex background containing environmental and genetic heterogeneities among humans, mouse models are useful tools to investigate the potential long-term effects of ART on offspring (Chen et al., 2014a; Vrooman and Bartolomei, 2017; Duranthon and Chavatte-Palmer, 2018). Studies using ART mouse models have demonstrated that IVF-ET offspring exhibit impaired glucose metabolism, including altered fasting glucose levels and impaired glucose tolerance (IGT) as adults (Calle et al., 2012; Chen et al., 2014a, b; Cerny et al., 2017; Vrooman and Bartolomei, 2017; Duranthon and Chavatte-Palmer, 2018). In contrast to IVF-ET, embryos are cryopreserved in liquid nitrogen, thawed and cultured in artificial medium during IVF-FET procedures. Although it is generally assumed that low temperature (−196°C) does not impair the developmental potential of embryos (Roque et al., 2013; Paulson, 2020), whether cryopreservation exacerbates the negative long-term impacts of IVF-ET and/or causes other adverse effects on offspring remains uncertain. The aim of this research was to investigate the long-term impact of IVF-FET on glucose and lipid metabolism compared with natural conception (NC) and IVF-ET in offspring fed a normal or high-fat diet (HFD) using mouse models. Mice offspring were divided into six groups according to the method of conception and the type of diet (NC-chow, IVF-chow, FET-chow, NC-HFD, IVF-HFD, and FET-HFD).

Our results found that IVF-FET conceived chow-fed male offspring displayed IGT and decreased insulin tolerance. Mice in the FET-HFD group displayed earlier and more severe IGT than those in the IVF-HFD compared with NC-HFD group. In the livers of mice in the FET-chow group, impaired hepatic insulin signaling suggested reduced glycogen synthesis (GCS), increased glycolysis and enhanced gluconeogenesis. Finally, we performed RNA-sequencing using livers harvested from offspring, and the results showed significantly downregulated expression of proteins involved in the insulin resistance pathway in the FET-chow group.

## Materials and Methods

### Animals and Experimental Design

All experimental procedures with mice were approved by the Shanghai Model Organisms Center’s Ethical Committee in Animal Research. Virgin 6- to 8-week-old B6D2F1/J (C57B6L/J × DBA2/J) female mice, adult B6D2F1/J males, 8-week-old ICR females and adult ICR vasectomized males were used. Pregnant mice were housed individually. The offspring were separated after weaning when about postnatal 21 days. Up to five mice with same sex were housed in each cage. All animals were kept in the same room maintained under a constant 12-h light/12-h dark cycle at 21–23°C with free access to food and water.

**FIGURE 1:**
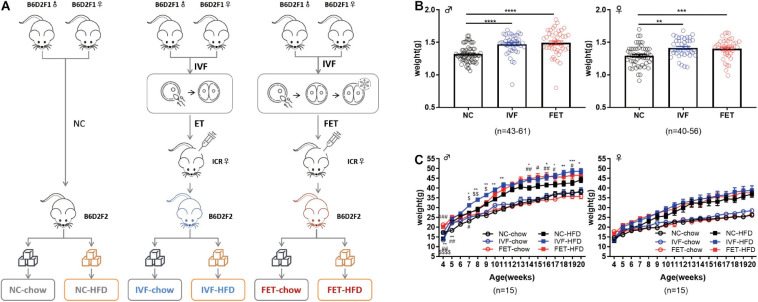
Experimental design, birthweights and growth curves of the offspring conceived by NC, IVF-ET and IVF-FET. **(A)** Experimental design. **(B)** Birthweights of male offspring (n_NC_ = 61, n_IVF_ = 43, and n_FET_ = 44) and female offspring (n_NC_ = 56, n_IVF_ = 40, and n_FET_ = 40). **(C)** Growth curves of male and female offspring after weaning (*n* = 15 mice per group). All data were expressed as the mean ± SEM. Symbols under curves: **P < 0.01 IVF-chow vs NC-chow; ^#^*P* < 0.05 FET-chow vs NC-chow; ^##^*P* < 0.01 FET-chow vs NC-chow; and ^$$$$^*P* < 0.0001 FET-chow vs IVF-chow. Symbols above the curves: *P < 0.05 IVF-HFD vs NC-HFD; **P < 0.01 IVF-HFD vs NC-HFD; ***P < 0.001 IVF-HFD vs NC-HFD; ^#^*P* < 0.05 FET-HFD vs NC-HFD; ^##^*P* < 0.01 FET-HFD vs NC-HFD; ^###^*P* < 0.001 FET-HFD vs NC-HFD; ^$^*P* < 0.05 FET-HFD vs IVF-HFD; and ^$$^*P* < 0.01 FET-HFD vs IVF-HFD (ANOVA).

The overall experimental design of this study is shown in the flowchart in Figure 1A. B6D2F1/J females and B6D2F1/J males were used to establish 2-cell embryos, as described below. Fresh 2-cell embryos and frozen-thawed 2-cell embryos were transferred to the oviducts of pseudopregnancy ICR mice, referred to as the IVF group and FET group, respectively. Additionally, we set up a natural pregnancy group as a control termed the NC group. On the day of birth, the pups were weighed. After weaning, the mice in each group were randomly divided into chow-fed or HFD-fed groups (starting at 4 weeks old for 16 weeks). HFD exposure (D12492, Fanbo, Shanghai, China) (nutrient composition 60% fat, 20% protein, and 20% carbohydrate) served as a “second hit” to unmask or amplify the underlying defects occurring in disease states.

### *In vitro* Fertilization

Female B6D2F1/J mice were super-ovulated with an intraperitoneal injection of 10 IU pregnant mare serum gonadotrophin (PMSG, Ningbo Second Hormone Factory, Ningbo, China), followed by 10 IU human chorionic gonadotrophin (HCG, Ningbo Second Hormone Factory, Ningbo, China) 48 h later. Oocytes were collected from the ampullae 13 h after HCG administration and fertilized *in vitro* with cauda epididymal sperm obtained from B6D2F1/J mice. Gametes were co-incubated in human tubal fluid medium (HTF, MR-070-D; Millipore, MA, United States) in a 5% CO_2_ and 5% O_2_ incubator at 37°C (MCO-5M, Panasonic, Gunma, Japan). After 15–16 h of culture, 2-cell embryos were washed with M2 medium (Millipore, MA, United States) three times for both embryo transfer and freezing.

### Frozen and Thawed Embryos

Mouse embryos in the 2-cell stage after completing the IVF protocol were frozen using a slow-freezing protocol (Testart et al., 1986). First, embryos were equilibrated in 0.75 mol/L propanediol (PROH) at room temperature for 10 min and then transferred to 1.5 mol/L PROH for 5 min. The embryos were finally loaded into plastic straws, which were placed in a controlled-rate freezer (Kryo 560-16, Planer Product Ltd., United Kingdom) and cooled from 20°C to −8°C at a rate of 2°C/min. After maintaining −8°C for 10 min, the temperature was slowly decreased to −3°C at a rate of −0.3°C/min and then rapidly reduced to −150°C at a rate of −50°C/min. Finally, the straws containing embryos were stored in liquid nitrogen.

After 7 days of cryopreservation, embryos were thawed using the following protocol. The straws were removed from liquid nitrogen, and after 30 s, the embryos were transferred to 1.0 mol/L PROH + 0.2 mol/L sucrose for 5 min and then 0.5 mol/L PROH + 0.2 mol/L sucrose for 5 min. Finally, embryos were placed in 0.3 mol/L sucrose for 10 min before being added to HTF medium. After thawing, 2-cell embryos were cultured in HTF medium for 2 h at 37°C in a 5% CO_2_ and 5% O_2_ incubator before embryo transfer.

### Embryo Transfer

Female ICR mice were used as pseudopregnant recipients for embryo transfer. Recipients were generated by mating with vasectomized male ICR mice. The day on which a plug was observed was considered to be E0.5 day of pseudopregnancy. Either fresh or frozen-thawed 2-cell embryos were surgically transferred to the fallopian tubes of pseudopregnant ICR females at E0.5. The mice were anesthetized by isoflurane. All recipient females were fed a chow diet.

### Glucose, Insulin and Pyruvate Tolerance Test

A glucose tolerance test (GTT) and pyruvate tolerance test (PTT) were performed at 8 am, following a 16-h fast, and an insulin tolerance test (ITT) was performed at 12 am, following an 8-h fast. Glucose (2 g/kg), pyruvate (2 g/kg) or insulin (0.75 IU/kg) was administrated intraperitoneally to mice. Glucose levels were measured using a blood glucose meter (Accu-Chek Performa; Roche Diagnostics) at 0, 15, 30, 60, and 120 min post injection. GTTs and ITTs were performed at 4, 8, 14, and 20 weeks old, and PTTs were performed at 20 weeks old. Fasting blood glucose (FBG) levels and fasting serum insulin (FINS) concentrations were measured at 8 am, following a 16-h fast. The female mice at were detected by GTT and ITT at the estrous phase.

### *In vivo* Glucose-Stimulated Insulin Secretion Test

Glucose-stimulated insulin secretion (GSIS) assays were performed by intraperitoneally administering glucose (2 g/kg) at 8 am, after a 16-h fast. Blood samples were collected from the tail tip at 0, 15, and 30 min after glucose administration. Insulin serum levels were determined using mouse insulin enzyme-linked immunosorbent assay (ELISA) kits (Crystal Chem, Downers Grove, IL, United States). Absorbance was monitored with a Synergy H1 microplate reader (BioTek Instruments, VT, United States). The Homeostasis Model Assessment-Insulin Resistance (HOMA-IR) index was calculated according to the formula: HOMA-IR = FBG (mg/dL) × FINS (μU/mL)/405.

### Lipid Parameters and Serum Testosterone Level Assessment

After a 4-h fast, blood samples were collected from the retro-orbital sinus puncture in anesthetized mice, and serum was stored at −80°C before analysis. Serum cholesterol (TC), triglyceride (TG), high-density lipoprotein (HDL), low-density lipoprotein (LDL), and non-esterified fatty acids (NEFAs) were assayed using a biochemical analyzer (FR120; Toshiba, Tokyo, Japan). The serum total and free testosterone levels were assessed by ELISA (CSB-E05101m and CSB-E05098m, Cusabio, Wuhan, China).

### Tissue Collection and Histological Analysis

Liver tissue was removed from 20-week-old mice after 4 h of fasting. The tissues were fixed in 4% paraformaldehyde for histological analysis or frozen in liquid nitrogen. Hematoxylin and eosin (H&E) staining and periodic acid–Schiff (PAS) staining were performed on paraffin sections after deparaffinization and rehydration to observe the morphometry. Oil red O (ORO) staining was used to stain neutral TG and lipids in frozen sections. All sections were imaged using a light microscope (Leica DMi8, Germany).

### Transcriptome Library Preparation and RNA-Seq

Total RNA was isolated from livers using TRIzol reagent (Invitrogen, Carlsbad, CA, United States). The quality of total RNA was confirmed by agarose gel electrophoresis, and RNA was used for the construction of a cDNA library. The sequencing of cDNA was conducted on an Illumina Nova6000 system and performed by Genergy biological technology Co., Ltd. (Shanghai, China). The reads were trimmed and then mapped to the entire genome. To analyze the glucose metabolic-related gene ontology (GO) terms and KEGG pathways, gene set enrichment analysis (GSEA) was performed using “clusterProfiler” (v3.14.3) in R (Yu et al., 2012). When the nominal *P* value was less than 0.05 and |NES| > 1, the enriched gene set in GSEA was statistically significant.

### Quantitative Real-Time PCR

Total RNA was extracted from livers using TRIzol reagent (Invitrogen, Carlsbad, CA, United States) and reverse transcribed using a PrimeScript RT reagent Kit with gDNA Eraser (RR047A, Takara, Japan). The TB green Premix EX Taq kit (RR420A, Takara, Japan) was used for PCR. Real-time PCR reactions were run on Applied Biosystems QuantStudio 7 Flex PCR systems (Thermo Fisher Scientific Inc., United States). The primer sequences were provided in Supplementary Table 1.

### Glucose Metabolism Enzyme Activity Assay

Liver tissues (0.1 g) were homogenized, and the activities of enzymes involved in glycogen synthesis (glycogen synthase, GCS); glycogenolysis (glycogen phosphorylase a, GPa); and gluconeogenesis (glucose 6 phosphatase, G6P and phosphoenolpyruvate carboxylase kinase, PEPCK) were evaluated by enzyme activity assay kits (GCS-1-Y, GPA-a-Y, G6P-1-Y, and PEPCK-1-Y; Comin; Suzhou, China) according to the manufacturer’s protocols.

### Western Blotting

Protein was extracted from liver tissues using RIPA lysis buffer (P0013B; Beyotime, Shanghai, China). Samples were separated using SDS-PAGE at 80–120 V for 90 min and transferred onto a PVDF membrane at 220 mA for 90 min. The antibodies were listed in Supplementary Table 2. Primary antibodies were incubated overnight at 4°C, and second antibodies were incubated for 1 h at room temperature. The signals were visualized using an enhanced chemiluminescence system (Amersham Imager 600, GE Healthcare Life Sciences, Pittsburgh, PA, United States).

### Statistical Analysis

All data were shown as the mean ± SEM. Statistical analysis was performed by a one-way ANOVA and Chi-squared test using SPSS 22.0 software. Tukey’s test was used as the *post hoc* test for ANOVA. The area under the curves (AUCs) of glucose levels in GTT, ITT, and PTT were calculated by GraphPad Prism 8.0. Because different glucose metabolism patterns occur in mice fed different diets, we did not compare groups exposed to different diets and only compared the results within the chow-fed and HFD-fed groups. A *P* value <0.05 was considered statistically significant.

## Results

### Distinct Body Weight Trajectories Among Offspring Fed a Normal Chow or HFD Conceived by NC, IVF-ET, and IVF-FET

Our experimental design is shown in Figure 1A. There was no significant difference in the pregnancy rates and embryo implantation rates between the IVF-ET and IVF-FET groups (Supplementary Table 3). The litter size was similar among the three groups (NC: 7.9 ± 1.5, IVF-ET: 7.5 ± 2.5, and IVF-FET: 8.4 ± 2.1). The sex ratio (male/female) was 1.1 (63:56) in the NC group, 1.1 (43:40) in the IVF group and 1.1 (44:40) in the FET group. However, the birthweights of offspring in both the IVF and FET groups were higher than those in the NC group at postnatal day 0 (Figure 1B). After weaning, the weights of male offspring, but not female offspring, remained significantly higher in the IVF-ET and IVF-FET groups than those in the NC group. However, no significant difference was detected after 8 weeks of age among the NC-chow, IVF-chow and FET-chow groups (Figure 1C). Importantly, the body weights of male offspring, but not female offspring challenged with a HFD after 4 weeks old, were increased in the IVF-HFD and FET-HFD groups compared with those in the NC-HFD group from 7-weeks-old onward. These results suggested that both IVF-ET and IVF-FET procedures interfered with the normal development of offspring in mice.

### Impaired Glucose Tolerance and Decreased Insulin Tolerance in Offspring in the FET-Chow Group

**FIGURE 2:**
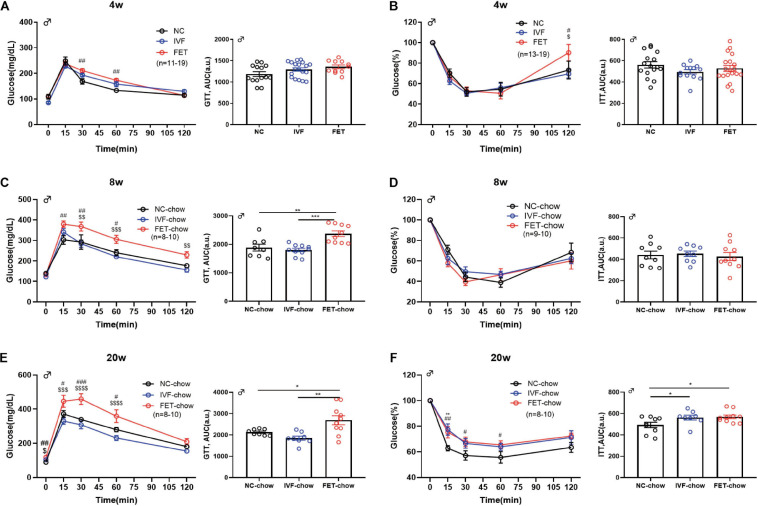
GTT and ITT in chow-fed male offspring. **(A)** Glucose tolerance test and AUC in 4-week-old chow-fed male offspring (n_NC–chow_ = 14, n_IVF–chow_ = 19, and n_FET–chow_ = 11). **(B)** Insulin tolerance test and AUC in 4-week-old chow-fed male chow offspring (n_NC–chow_ = 16, n_IVF–chow_ = 13, and n_FET–chow_ = 19). **(C)** Glucose tolerance test and AUC in 8-week-old chow-fed male offspring (n_NC–chow_ = 8, n_IVF–chow_ = 10, and n_FET–chow_ = 10). **(D)** Insulin tolerance test and AUC in 8-week-old chow-fed male offspring (n_NC–chow_ = 9, n_IVF–chow_ = 9, and n_FET–chow_ = 10). **(E)** Glucose tolerance test and AUC in 20-week-old chow-fed male offspring (n_NC–chow_ = 8, n_IVF–chow_ = 8, and n_FET–chow_ = 10). **(F)** Insulin tolerance test and AUC in 20-week-old chow-fed male offspring (n_NC–chow_ = 9, n_IVF–chow_ = 8, and n_FET–chow_ = 10). All data were expressed as the mean ± SEM. **P < 0.01 IVF-chow vs NC-chow; ^#^*P* < 0.05 FET-chow vs NC-chow; ^##^*P* < 0.01 FET-chow vs NC-chow; ^###^*P* < 0.001 FET-chow vs NC-chow; ^$^*P* < 0.05 FET-chow vs IVF-chow; ^$$^*P* < 0.01 FET-chow vs IVF-chow; and ^$$$^*P* < 0.001 FET-chow vs IVF-chow (ANOVA).

To further investigate the effects of IVF-ET and IVF-FET on glucose homeostasis, we performed GTTs and ITTs in male IVF and IVF-FET offspring and NC offspring fed a normal chow. Figures 2A,C,E show the GTT results of male offspring at the age of 4, 8, and 20 weeks, respectively. Although there was no difference in the AUC among the three chow diet groups, the blood glucose level in the offspring fed a normal chow in the FET-chow group was higher at 30 and 60 min post glucose injection at 4 weeks compared with that in the NC-chow and IVF-chow groups. Furthermore, a higher GTT AUC in the FET-chow group was observed at the age of 8 and 20 weeks. These results suggested IGT in the offspring in the FET-chow group. In addition, the ITT results (Figures 2B,D,F) showed that insulin sensitivity was decreased in 20-week-old offspring in the IVF-chow and FET-chow groups. The GTT and ITT results of female offspring were presented in Supplementary Figure 1. The GTT AUCs of female offspring conceived by IVF-ET and IVF-FET and fed a normal chow were comparable to those of the female offspring in the NC group before 14 weeks of age. Up to 20-weeks-old, the female offspring in the FET-chow group displayed impaired glucose homeostasis compared with the NC-chow and IVF-chow groups. No significant differences were observed in the ITT results among female offspring fed a normal chow in the three groups. Collectively, these results suggested that the offspring conceived by FET were predisposed to glucose metabolism disorders.

### Earlier and More Severe Impaired Glucose Intolerance Onset in the Offspring in the FET-HFD Group Compared With the IVF-HFD Group

**FIGURE 3:**
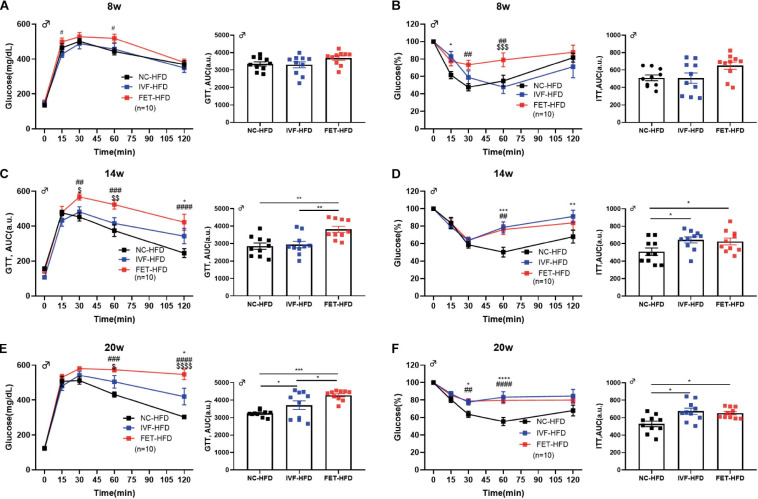
GTT and ITT in HFD-fed male offspring. **(A)** Glucose tolerance test and AUC in 8-week-old HFD-fed male offspring (*n* = 10 mice per group). **(B)** Insulin tolerance test and AUC in 8-week-old HFD-fed male chow offspring (*n* = 10 mice per group). **(C)** Glucose tolerance test and AUC in 14-week-old HFD-fed male offspring (*n* = 10 mice per group). **(D)** Insulin tolerance test and AUC in 14-week-old HFD-fed male offspring (*n* = 10 mice per group). **(E)** Glucose tolerance test and AUC in 20-week-old HFD-fed male offspring (*n* = 10 mice per group). **(F)** Insulin tolerance test and AUC in 20-week-old HFD-fed male offspring (*n* = 10 mice per group). All data were expressed as the mean ± SEM. *P < 0.05 IVF-HFD vs NC-HFD; **P < 0.01 IVF-HFD vs NC-HFD; ***P < 0.001 IVF-HFD vs NC-HFD; ^#^*P* < 0.05 FET-HFD vs NC-HFD; ^##^*P* < 0.01 FET-HFD vs NC-HFD; ^###^*P* < 0.001 FET-HFD vs NC-HFD; ^####^*P* < 0.0001 FET-HFD vs NC-HFD; ^$^*P* < 0.05 FET-HFD vs IVF-HFD; ^$$^*P* < 0.01 FET-HFD vs IVF-HFD; ^$$$^*P* < 0.001 FET-HFD vs IVF-HFD; and ^$$$$^*P* < 0.0001 FET-HFD vs IVF-HFD (ANOVA).

We further assessed the influence of IVF-ET and IVF-FET on offspring challenged with metabolic stress (Figures 3A,C,E). No significant difference in the GTT AUC at 8 weeks was found in the offspring fed a HFD in any of the groups. At 14 weeks, the GTT AUC in the offspring of the FET-HFD group was higher than that of the NC-HFD and IVF-HFD groups. At 20 weeks, the GTT AUC in both the IVF-HFD and FET-HFD groups was higher than that in the NC-HFD group. Of note, the GTT AUC for the offspring in the FET-HFD group was much higher than that in the IVF-HFD group. Moreover, the ITT AUC was increased in the offspring of the IVF-FET group compared with the other HFD-fed groups at 8-weeks-old (Figure 3B). At 14 and 20-weeks-old, the ITT AUC in the offspring of the IVF-HFD group was comparable to that in the FET-HFD group, which was substantially higher than the ITT AUC in the NC-HFD group (Figures 3D,F). In contrast to the glucose metabolism status in female offspring conceived by IVF-ET and IVF-FET, we found that the GTT AUCs of female offspring conceived by IVF-ET and IVF-FET under metabolic stress were evidently higher than those in the offspring of the NC-HFD group at both 8 and 20-weeks-old (Supplementary Figure 1), although the insulin sensitivities were similar.

### Insulin Resistance and Abnormal Pyruvate Tolerance in the Offspring of the FET-Chow Group

**FIGURE 4:**
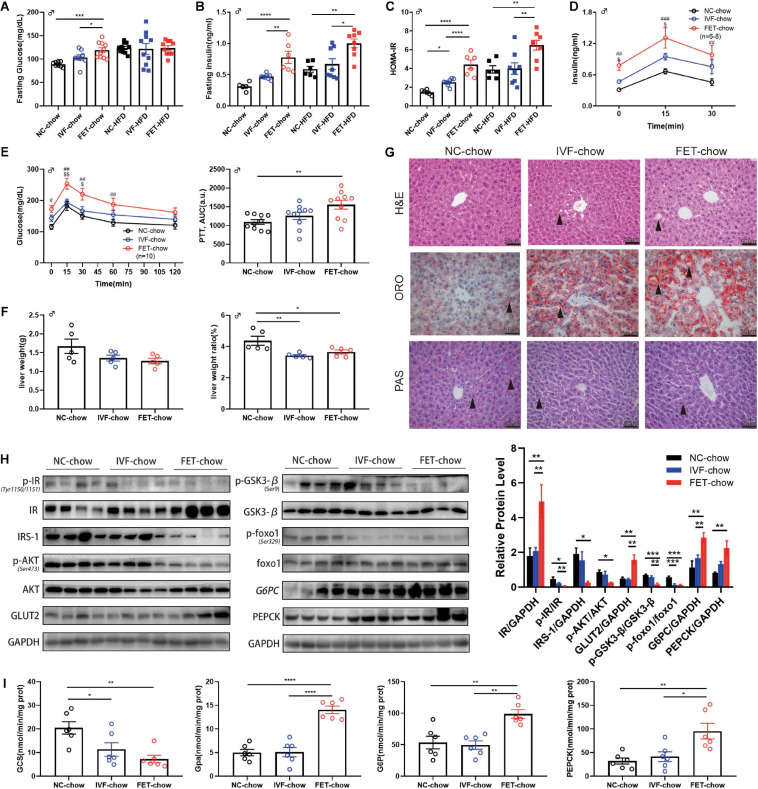
Insulin resistance and impaired hepatic insulin signaling in offspring of the FET-chow group. **(A)** Serum fasting glucose level in male offspring at 20 weeks old (n_NC–chow_ = 8, n_IVF–chow_ = 8, n_FET–chow_ = 10, n_NC–HFD_ = 10, n_IVF–HFD_ = 10, and n_FET–HFD_ = 10). **(B)** Serum fasting insulin level in male offspring at 20 weeks old (n_NC–chow_ = 6, n_IVF–chow_ = 8, n_FET–chow_ = 6, n_NC–HFD_ = 6, n_IVF–HFD_ = 8, and n_FET–HFD_ = 8). **(C)** HOMA-IR index in male offspring at 20 weeks old (n_NC–chow_ = 6, n_IVF–chow_ = 8, n_FET–chow_ = 6, n_NC–HFD_ = 6, n_IVF–HFD_ = 8, and n_FET–HFD_ = 8). **(D)** *In vivo* glucose-stimulated insulin secretion test in chow-fed male offspring at 20 weeks old (n_NC–chow_ = 6, n_IVF–chow_ = 8, and n_FET–chow_ = 6). **(E)** Pyruvate tolerance test and AUC in 20-week-old chow-fed male offspring (*n* = 10 mice per group). **(F)** Liver weights and liver weight/body weight ratios in chow-fed male offspring at 20 weeks old (*n* = 5 mice per group). **(G)** Liver morphology, H&E, ORO, and PAS staining (scale bar = 50 (μm). **(H)** Representative protein levels in chow-fed male offspring livers at 20 weeks old (*n* = 4 mice per group). **(I)** Enzymes activities in chow-fed male offspring livers at 20 weeks old (*n* = 6 mice per group). All data were expressed as the mean (SEM ^#^*P* < 0.05 FET-chow vs NC-chow; ^##^*P* < 0.01 FET-chow vs NC-chow; ^###^*P* < 0.001 FET-chow vs NC-chow; ^$^*P* < 0.05 FET-chow vs IVF-chow; and ^$$^*P* < 0.01 FET-chow vs IVF-chow (ANOVA).

Because male mice displayed a more obvious phenotype compared with females, we examined the former in greater detail. Figures 4A,B shows the impaired fasting glucose (IFG) and hyperinsulinemia at 20 weeks in the male offspring of the FET-chow group. The offspring fed a normal chow in the IVF-FET group displayed the highest HOMA-IR index. Meanwhile, the HOMA-IR index for offspring in the IVF-chow group was higher than that in the NC-chow group (Figure 4C). Similar results were observed among the offspring fed a HFD (Figure 4C). To determine whether insulin secretion was deficient, we examined serum insulin levels after intraperitoneal glucose injection. At 15 and 30 min after glucose injection, the insulin levels in the FET-chow group were significantly higher than those in the NC-chow group (Figure 4D). This result showed that the impaired metabolic homeostasis was not mainly caused by a deficiency in insulin secretion. The PTT is a method used to measure hepatic gluconeogenesis after an intraperitoneal injection of pyruvate. The PTT curve showed that 20-week-old offspring in the FET-chow group had evidently higher blood glucose levels than those in the NC-chow group at 0, 15, 30, and 60 min after glucose injection, whereas the curve for the offspring in the IVF-chow group was similar to that in the NC-chow group (Figure 4E). The results of the PTT and ITT revealed liver glucose regulatory dysfunction in the offspring of the FET-chow group compared with offspring in the NC-chow and IVF-chow groups at 20 weeks old.

### Impaired Hepatic Glucose Metabolism and Insulin Signaling in the Offspring of the FET-Chow Group

At 20 weeks, we measured the weight of organs associated with glucose and lipid metabolism, such as the liver, pancreas, rectus femoris quadriceps muscle and epididymal fat, and calculated the relative organ weight/body weight ratio in male offspring (Figure 4F and Supplementary Figure 2). A reduced liver weight ratio was observed in both the IVF-chow and FET-chow groups. H&E staining revealed hepatic ballooning degeneration (black arrows) in the livers from the male offspring in the IVF-chow and FET-chow groups (Figure 4G). In addition, we observed fewer glycogen storages (black arrows) in the livers from the male offspring in both the IVF-chow and FET-chow groups using PAS staining (Figure 4G). The PI3K/AKT signaling pathway, one of the major downstream regulators in response to insulin, has been shown to play an important role in the regulation of hepatic glucose homeostasis, including the inhibition of gluconeogenesis and glycogenolysis pathways and the activation of glycolysis pathways (Yu et al., 2008). Glucose transporter 2 (Glut2) is a crucial trans-membrane transporter protein that transports glucose between the liver and blood. Because abnormal PI3K/AKT signaling is associated with insulin resistance, we determined the expression levels of key proteins involved in this pathway in the livers of offspring by western blotting (Figure 4H). In FET-chow livers, expression of the insulin receptor (IR) was increased, whereas phosphorylation of the IR (p-IR) at Tyr1150/1151 was significantly decreased compared with that in the offspring from the NC-chow group. Meanwhile, decreased insulin receptor substrate-1 (IRS-1), reduced phosphorylation of Akt (p-Akt) at Ser473 and increased Glut2 were detected in the livers of offspring fed a normal chow from the IVF-FET group. In the FET-chow group, Gsk3-β phosphorylation (p-Gsk3-β) at Ser9 and Foxo1 phosphorylation (p-Foxo1) at Ser329 were significantly decreased, indicating reduced GCS and increased glycolysis. Furthermore, we detected the activity of the rate-limiting enzymes of GCS and glycolysis (Gpa) using a spectrophotometer. Similar to the western blotting results, hepatic GCS activity was decreased, and Gpa activity was increased in the offspring of the FET-chow group compared with the offspring from both the IVF-ET and NC groups (Figure 4I). G6pc and Pepck are two key enzymes in gluconeogenesis. We found that the protein expression levels of both G6pc and Pepck were significantly upregulated in the FET-chow group (Figure 4H), and the activities of these two enzymes were increased, indicating an enhanced gluconeogenesis status (Figure 4I) compared with that in the NC-chow group, similar to the results of PTT assays.

### Abnormal Lipid Profile in the Offspring of IVF-Chow and FET-Chow Groups

The liver is not only the central hub of glucose metabolism but also an important organ in lipid metabolism. Mice offspring at the age of 20 weeks, but not 8 weeks, in both IVF-chow and FET-chow groups exhibited higher serum TG, LDL and NEFA levels and lower HDL levels compared with those in the NC-chow group, indicating dyslipidemia (Table 1). In addition, the HDL levels in the FET-chow group were lower than those in the IVF-chow group. Furthermore, lipid accumulation was clearly detected in the livers of the offspring from the IVF-chow and FET-chow groups via ORO staining assays (Figure 4G).

**TABLE 1:** Lipid profile of chow-fed male offspring at 8 and 20 w.

	8 w			20 w		
	NC-chow (*n* = 7)	IVF-chow (*n* = 7)	FET-chow (*n* = 7)	NC-chow (*n* = 7)	IVF-chow (*n* = 7)	FET-chow (*n* = 7)
TC (mmol/L)	3.18 ± 0.15	3.16 ± 0.12	3.20 ± 0.12	4.19 ± 0.19	3.82 ± 0.31	4.15 ± 0.33
TG (mmol/L)	1.44 ± 0.06	1.52 ± 0.12	1.44 ± 0.06	1.61 ± 0.08	1.98 ± 0.09*	2.08 ± 0.15^##^
HDL (mmol/L)	2.29 ± 0.10	2.29 ± 0.09	2.22 ± 0.09	2.84 ± 0.10	2.47 ± 0.10**	2.13 ± 0.07^####[*dollar*]^
LDL (mmol/L)	0.25 ± 0.03	0.29 ± 0.01	0.27 ± 0.01	0.23 ± 0.03	0.31 ± 0.02*	0.34 ± 0.01^##^
NEFA (mmol/L)	1.60 ± 0.18	1.64 ± 0.13	1.98 ± 0.19	1.27 ± 0.12	1.87 ± 0.12**	1.67 ± 0.09#

TC, total cholesterol; TG, triacylglycerol; HDL, high-density lipoprotein; LDL, low-density lipoprotein; NEFA, non-esterified fatty acids. All data were expressed as mean ± SEM *P < 0.05 vs NC-chow, **P < 0.01 vs NC-chow. ^#^*P* < 0.05 vs NC-chow, ^##^*P* < 0.01 vs NC-chow, ^####^*P*<0.0001 vs NC-chow, ^$^*P*<0.05 vs IVF-chow. Significance was determined by one-way ANOVA.

### Alterations in the Hepatic Transcriptome in Offspring of the IVF-Chow and FET-Chow Groups

**FIGURE 5:**
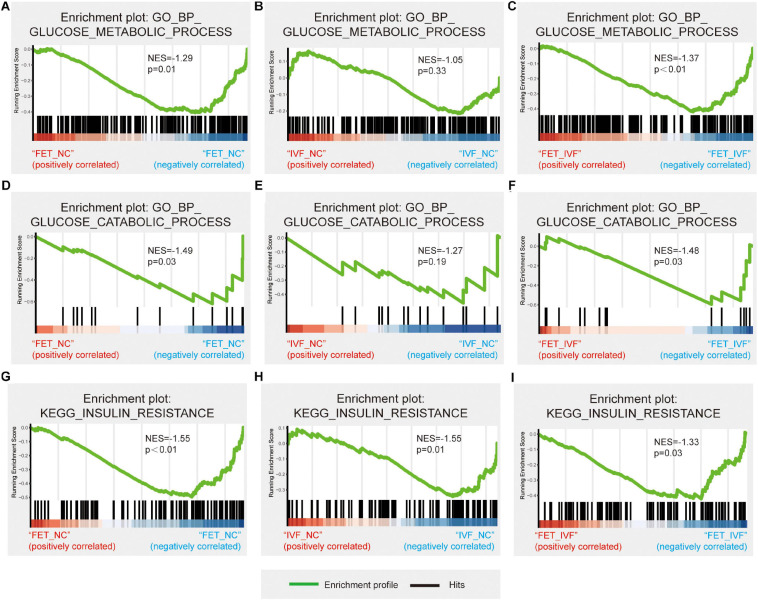
Hepatic transcriptome changes in glucose metabolism in IVF-chow and FET-chow groups. **(A–C)** Gene set enrichment plots related to glucose metabolic processes in the FET-chow group compared with the NC-chow group, the IVF-chow group compared with the NC-chow group and the FET-chow group compared with the IVF-chow group. **(D–F)** Gene set enrichment plots related to glucose catabolic processes in the FET-chow group compared with the NC-chow group, the IVF-chow group compared with the NC-chow group and the FET-chow group compared with the IVF-chow group. **(G–I)** Gene set enrichment plots related to the insulin resistance pathway in the FET-chow group compared with the NC-chow group, the IVF-chow group compared with the NC-chow group and the FET-chow group compared with the IVF-chow group. NES, normalized enrichment score; GO, Gene Ontology; BP, biological process; KEGG, Kyoto Encyclopedia of Genes and Genomes. (*n* = 3 mice per group).

To explore the underlying mechanisms of the aberrant metabolism in male offspring conceived by IVF and FET, we performed the RNA-sequencing of livers from three groups. To confirm the accuracy of the sequencing data, four randomly selected mRNAs related to glucose metabolism were validated by qPCR (Supplementary Figure 3). The qPCR results revealed the same trends as those in the sequencing dataset. After a pairwise comparison of the GSEA, we found different liver transcriptome statuses in the IVF-chow and FET-chow groups. Compared with the NC-chow and IVF-chow groups, genes involved in glucose metabolic processes and glucose catabolic processes were significantly downregulated in the FET-chow group (Figures 5A,C,D,F). However, there was no significant difference in these two processes in the correlation in the IVF-chow group compared with the NC-chow group (Figures 5B,E; *P* > 0.05). These results suggested impaired glucose metabolic and catabolic processes in the livers from the FET-chow group compared with those in the IVF-chow and NC-chow groups.

As for KEGG analysis, the insulin resistance pathway was significantly down-regulated in the livers from both the IVF-chow and FET-chow groups compared with the NC-chow group (Figures 5G,H). Additionally, compared with the IVF-chow group, the pathway was downregulated in the livers from the FET-chow group (Figure 5I). These results suggested more severe alterations in the expression of genes involved in this pathway in the FET-chow group than that in the IVF-chow group.

## Discussion

In agreement with the “gamete and embryo-fetal origins of adult diseases” theory (Barker et al., 1989; Zou et al., 2019), our research revealed long-term influences of glucose and lipid metabolism on IVF-FET-conceived male mice. Previous studies have used animal models to explore the short-term effects in offspring conceived by IVF-FET. In a rabbit model, the offspring generated by cryopreservation embryo transfer exhibited impaired body growth during the observation time (Garcia-Dominguez et al., 2020). Another study observed increased bodyweight in senescent mice born from cryopreserved embryos (Dulioust et al., 1995). However, a comprehensive understanding of the long-term effects and possible health concerns in FET offspring remains to be established.

In our mice conceived by FET, there were three important findings. First, more severe glucose metabolism dysfunction was observed in the offspring fed either a normal chow or HFD diet in the IVF-FET group than in offspring from the IVF-ET group. Furthermore, the offspring in the FET-chow group showed an abnormal lipid profile. Second, insulin resistance was the primary manifestation in the IVF-FET mouse model, and the liver played an important role in this metabolic dysfunction. Third, more dramatic alterations at the transcriptomic level in several genes involved in glucose metabolism were observed in the livers of offspring conceived by IVF-FET than in those in the IVF-ET group, which was consistent with the glucose metabolic phenotypes. These findings directly demonstrated that the preimplantation embryo cryopreservation and thawing processes, in combination with the artificial environments during fertilization to the 2-cell stage, lead to aberrant glucose and lipid metabolism in the offspring conceived by FET-ET.

As mentioned in the results, chow-fed male offspring in the IVF and FET groups showed higher birth weights, which reached a similar level as the NC group after 8 weeks. Previously published studies indicated that IVF mice offspring exhibited low birth weights and catch-up growth (Chen et al., 2014b; Feuer et al., 2014). This contradiction with our results is partly due to the different mouse strains and culture media used (Donjacour et al., 2014).

Sex-specific phenotypes were found in our study. As mentioned above, female offspring in the FET-chow group exhibited IGT at 20-weeks-old without IFG or decreased insulin tolerance. It appeared that the phenotypes in females were less severe than those observed in male offspring from the FET-chow group. Some studies have reported severe glucose metabolism dysfunction in female offspring, whereas others indicated that this occurred in male offspring only (Scott et al., 2010; Calle et al., 2012; Chen et al., 2014b; Donjacour et al., 2014; Feuer et al., 2014).

Previous studies have demonstrated that androgen/AR signaling is involved in regulating metabolic homeostasis and the men with lower testosterone level in serum are predispose to metabolic disorders (Yu et al., 2014). Testosterone supplementation is benefit to glucose homeostasis in male mouse model of type 2 diabetes mellitus (Pal and Gupta, 2016). However, we do not find any significant differences of testosterone levels in serum among three groups (Supplementary Figures 4A,B). And no significant correlation between total or free testosterone levels in serum and HOMA-IR index is observed (Supplementary Figures 4C,D). Moreover, there is no apparent alteration of testosterone levels in serum from the male mice offspring conceived by FET at age of 20 weeks old. Therefore, we reason that the observed sex differences in our study do not cause by testosterone defects.

We assessed serum fasting glucose, insulin level and hepatic relative pAKT protein expression in mice at 4-weeks-old. No significant differences were observed (Supplementary Figure 5). This strongly suggested the abnormality of glucose metabolism might occur depending on age. In our mice conceived by FET, chow-fed male offspring showed IGT after 8-weeks-old compared with NC-chow and IVF-chow offspring. Decreased insulin tolerance and IFG appeared at 20-weeks-old in chow-fed male offspring from the FET-ET group. After a “second hit” with the HFD, the offspring conceived by IVF-FET showed earlier and more severe IGT onset than those from the IVF-HFD group. We suggest that the insulin resistance was associated with glucose metabolism disorders in these offspring. Then, we detected the expression of key proteins involved in the PI3K/AKT pathway in the liver as this pathway is the primary regulator of insulin resistance. The elevated expression of Glut2 in FET-chow mice indicated an active transport status between the blood and liver. In addition, the increase in glycogenolysis and gluconeogenesis and decrease in GCS indicated decreased glycogen levels in the liver. The results of GO enrichment analysis revealed a downregulated transcriptional status of genes involved in glucose metabolic and catabolic processes in the livers from FET-chow mice compared with those from NC-chow and IVF-chow mice. The insulin resistance status in FET-chow mice was consistent with the liver transcriptome KEGG results. We also observed increased hepatic lipid deposition, indicating abnormal lipid metabolism. It is well known that hyperglycemia, hyperinsulinemia, insulin resistance, and hypertriglyceridemia are the main characteristics of type 2 diabetes (T2D) and other metabolic syndromes. Therefore, we speculate that IVF-FET conceived male mice are predisposed to T2D.

Importantly, we only used a slow-freezing protocol. Although slow-freezing methods have been used clinically for several years, this technique has been gradually replaced by vitrification. According to a systematic and meta-analysis review, vitrification achieves higher clinical pregnancy rates and live birth rates compared with slow-freezing (Rienzi et al., 2017). One study on human embryos suggested that vitrification affects the DNA integrity of embryos to a much lesser extent than slow freezing (Martinez-Burgos et al., 2011). Therefore, whether different embryo freezing methods alter offspring glucose metabolism patterns still needs to be determined in a future study.

Our mouse model eliminated the potential complex background in humans, and using this model, we determined the long-term effects of cryopreservation. However, due to the complex genetic environment, hormone environment and administration of various ovulation drugs in humans, our conclusion may not be directly applicable to humans. Therefore, more experimental and clinical studies are needed. It is well established that insulin resistance has a causal role in T2D, and early diagnosis and intervention are critical to prevent T2D and delay or even prevent the serious complications associated with diabetes (Grundy, 2012). In adults with IGT, interventions may be required to alter the progressive β-cell dysfunction (RISE Consortium, 2019) and even reduce the risk of T2D (DeFronzo et al., 2011). If humans born from FET are at high risk of IGT or insulin resistance, then early detection and intervention are necessary to prevent or delay T2D and its associated complications. This study has important implications for both endocrinologists and ART physicians.

The mechanism underlying the phenotypes of ART-conceived offspring is not entirely clear, but epigenetic changes are a well-excepted contributor. The fertilization and preimplantation stages are critical periods of development when genome-wide reprogramming occurs. A previous study suggested that during cryopreservation, 2-cell mouse embryos are subjected to physical and chemical alterations, including the destruction of cell membrane integrity, redistribution of actin fibers, mitochondrial depolarization and increased reactive oxygen species production (Ahn et al., 2002). Active division in cells might make their genetic apparatus more vulnerable to the insult of extreme factors, such as cryopreservation (Kopeika et al., 2015). The inheritance of aberrant epigenetic modifications also might involve direct effects, intergenerational effects and transgenerational effects in offspring.

Generally, the FET embryos suffer cooling damage in freezing step and thermal shocking in thawing step. According to previous studies, cooling velocity, the type of non-permeating cryoprotectant agents and thawing velocity could cause varying degrees of cellular and zona damages including chromatin damage, altered mitochondrial distribution pattern, biological membranes damage and ice crystals damage (Miyamoto and Ishibashi, 1978; Ashwood-Smith et al., 1988; Mazur, 1990; Titterington and Robinson, 1996; Somoskoi et al., 2015). Especially, the damages from ice crystals are produced in both cooling and thawing steps (Mazur, 1990). It has known that the damages caused by ice crystals during embryo frozen and thawed processes can reduce the developmental potentials of preimplantation embryos (Titterington and Robinson, 1996). Actually, it is difficult to distinguish the damages caused by the whole freezing technology or the thermal shock in the thawing step. Further research involving the mechanisms on how embryo cryopreservation alters glucose metabolism and the liver transcriptome in adulthood are needed.

## Conclusion

Our study demonstrated a novel correlation between preimplantation embryo cryopreservation and glucose metabolism dysfunction mainly characterized by insulin resistance in offspring using various mouse models. This is the first detailed description of glucose metabolism in IVF-FET-conceived male mouse offspring. For the first time, our study reported an increased risk of metabolic disorders in IVF-FET conceived offspring compared with those born after IVF-ET. Although more clinical trials and basic science studies are required, our findings provide valuable information for preventive and clinical decisions. We are currently working on optimizing embryo freezing protocols to minimize unwanted effects. This study is just the first step in this direction, and more efforts are needed.

## Data Availability Statement

The datasets presented in this study can be found in online repositories. The names of the repository/repositories and accession number(s) can be found below: https://www.ncbi.nlm.nih.gov/geo/, GSE164819.

## Ethics Statement

The animal study was reviewed and approved by Shanghai Model Organisms Center’s Ethical Committee in Animal Research.

## Author Contributions

NQ and ZZ designed and performed the experiments and analyzed the data. NQ, ZZ, and WZ wrote and edited the manuscript. KZ and WS performed bioinformatics analysis. CY, XL, ZD, and YM contributed to perform experiment. XL, JS, and GD contributed to study design, discussion, and conducted experiments. YW and HH designed and supervised the research, contributed to discussion, and edited the manuscript. HH was the guarantor of this work and, as such, had full access to all data in the study and took responsibility for the integrity and accuracy of data analysis.

## Conflict of Interest

The authors declare that the research was conducted in the absence of any commercial or financial relationships that could be construed as a potential conflict of interest.

## Supplementary Material

Supplementary Figure 1 GTT and ITT in female offspring. **(A)** Glucose tolerance test and AUC in 4-week-old female offspring (*n* = 10 mice per group). **(B)** Insulin tolerance test and AUC in 4-week-old female offspring (*n* = 10 mice per group). **(C)** Glucose tolerance test and AUC in 8-week-old female offspring (n_NC–chow_ = 7, n_IVF–chow_ = 10, n_FET–chow_ = 10, n_NC–HFD_ = 8, n_IVF–HFD_ = 8, and n_FET–HFD_ = 10). **(D)** Insulin tolerance test and AUC in 8-week-old female offspring (*n* = 10 mice per group). **(E)** Glucose tolerance test and AUC in 14-week-old female offspring (n_NC–chow_ = 10, n_IVF–chow_ = 9, n_FET–chow_ = 10, n_NC–HFD_ = 10, n_IVF–HFD_ = 10, and n_FET–HFD_ = 10). **(F)** Insulin tolerance test and AUC in 14-week-old female offspring (n_NC–chow_ = 10, n_IVF–chow_ = 10, n_FET–chow_ = 10, n_NC–HFD_ = 9, n_IVF–HFD_ = 10, and n_FET–HFD_ = 10). **(G)** Glucose tolerance test and AUC in 20-week-old female offspring (n_NC–chow_ = 9, n_IVF–chow_ = 10, n_FET–chow_ = 10, n_NC–HFD_ = 10, n_IVF–HFD_ = 10, and n_FET–HFD_ = 10). **(H)** Insulin tolerance test and AUC in 20-week-old female offspring (n_NC–chow_ = 9, n_IVF–chow_ = 10, n_FET–chow_ = 10, n_NC–HFD_ = 10, n_IVF–HFD_ = 10, and n_FET–HFD_ = 10). All data were expressed as the mean ± SEM. Symbols under curves: ^∗^*P* < 0.05 IVF-chow vs NC-chow; ^∗∗^*P* < 0.01 IVF-chow vs NC-chow; ^****^*P* < 0.0001 IVF-chow vs NC-chow; ^#^*P* < 0.05 FET-chow vs NC-chow; and ^$^*P* < 0.05 FET-chow vs IVF-chow. Symbols above the curves: ^#^*P* < 0.05 FET-HFD vs NC-HFD; ^##^*P* < 0.01 FET-HFD vs NC-HFD; ^###^*P* < 0.001 FET-HFD vs NC-HFD; ^$^*P* < 0.05 FET-HFD vs IVF-HFD; ^$$^*P* < 0.01 FET-HFD vs IVF-HFD; and ^$$$^*P* < 0.001 FET-HFD vs IVF-HFD (ANOVA).

Supplementary Figure 2 Organ weights and ratios in chow-fed male offspring. **(A–C)** Pancreas, rectus femoris muscle, and epididymal fat weights in chow-fed male offspring at 20 weeks old (*n* = 5 mice per group). **(D–F)** Pancreas, rectus femoris muscle, and epididymal fat weight/body weight ratios in chow-fed male offspring at 20 weeks old (*n* = 5 mice per group). All data were expressed as the mean ± SEM. male offspring at 20 weeks old (*n* = 5 mice per group). All data were expressed as the mean ± SEM.

Supplementary Figure 3 The qPCR validation of differential expression genes in sequencing data. Relative mRNA expression levels in the livers of offspring at 20 weeks old (*n* = 4 mice per group). All data were expressed as the mean ± SEM.

Supplementary Figure 4 The serum testosterone levels in male offspring. **(A)** Serum total testosterone level in offspring at 20 weeks old (*n* = 6 mice per group). **(B)** Serum free testosterone level in offspring at 20 weeks old (*n* = 6 mice per group). **(C)** The correlation between serum total testosterone and HOMA-IR in male offspring at 20 weeks old (*n* = 18). **(D)** The correlation between serum free testosterone and HOMA-IR in male offspring at 20 weeks old (*n* = 18). All data were expressed as the mean ± SEM.

Supplementary Figure 5 The serum fasting glucose, insulin level and relative pAKT/AKT protein expression in 4-week-old offspring. **(A)** Serum fasting glucose level in offspring at 4 weeks old (*n* = 6 mice per group). **(B)** Serum fasting insulin level in offspring at 4 weeks old (*n* = 6 mice per group). **(C)** HOMA-IR index in offspring at 4 weeks old (*n* = 6 mice per group). **(D)** Relative pAKTn chow-fed male offspring livers at 20 weeks old (*n* = 4 mice per group). All data were expressed as the mean ± SEM.

Supplementary Material The entire-unmodified gels related to Figure 4H.
