# Supplementary figures and images for: Abnormal Glucose Metabolism in Male Mice Offspring Conceived by in vitro Fertilization and Frozen-Thawed Embryo Transfer
Source: Front Cell Dev Biol. 2021 Feb 9;9:637781. doi: 10.3389/fcell.2021.637781 (PMC7900417; doi:10.3389/fcell.2021.637781)

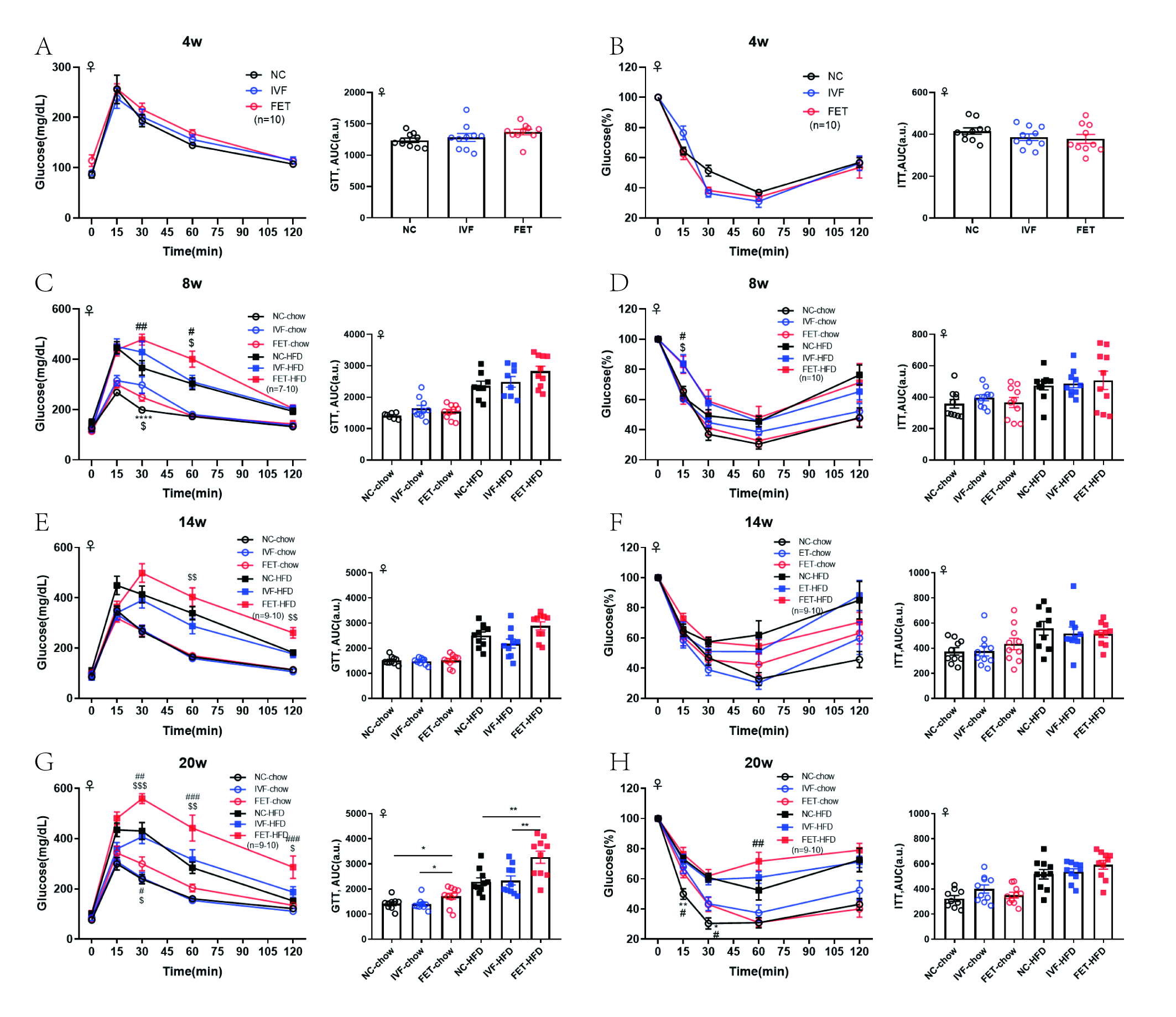

Supplement: Supplementary Figure 1 — GTT and ITT in female offspring. (A) Glucose tolerance test and AUC in 4-week-old female offspring (n = 10 mice per group). (B) Insulin tolerance test and AUC in 4-week-old female offspring (n = 10 mice per group). (C) Glucose tolerance test and AUC in 8-week-old female offspring (nNC–chow = 7, nIVF–chow = 10, nFET–chow = 10, nNC–HFD = 8, nIVF–HFD = 8, and nFET–HFD = 10). (D) Insulin tolerance test and AUC in 8-week-old female offspring (n = 10 mice per group). (E) Glucose tolerance test and AUC in 14-week-old female offspring (nNC–chow = 10, nIVF–chow = 9, nFET–chow = 10, nNC–HFD = 10, nIVF–HFD = 10, and nFET–HFD = 10). (F) Insulin tolerance test and AUC in 14-week-old female offspring (nNC–chow = 10, nIVF–chow = 10, nFET–chow = 10, nNC–HFD = 9, nIVF–HFD = 10, and nFET–HFD = 10). (G) Glucose tolerance test and AUC in 20-week-old female offspring (nNC–chow = 9, nIVF–chow = 10, nFET–chow = 10, nNC–HFD = 10, nIVF–HFD = 10, and nFET–HFD = 10). (H) Insulin tolerance test and AUC in 20-week-old female offspring (nNC–chow = 9, nIVF–chow = 10, nFET–chow = 10, nNC–HFD = 10, nIVF–HFD = 10, and nFET–HFD = 10). All data were expressed as the mean ± SEM. Symbols under curves: ∗P < 0.05 IVF-chow vs NC-chow; ∗∗P < 0.01 IVF-chow vs NC-chow; ****P < 0.0001 IVF-chow vs NC-chow; #P < 0.05 FET-chow vs NC-chow; and $P < 0.05 FET-chow vs IVF-chow. Symbols above the curves: #P < 0.05 FET-HFD vs NC-HFD; ##P < 0.01 FET-HFD vs NC-HFD; ###P < 0.001 FET-HFD vs NC-HFD; $P < 0.05 FET-HFD vs IVF-HFD; $$P < 0.01 FET-HFD vs IVF-HFD; and $$$P < 0.001 FET-HFD vs IVF-HFD (ANOVA). [file Image_1.TIF]

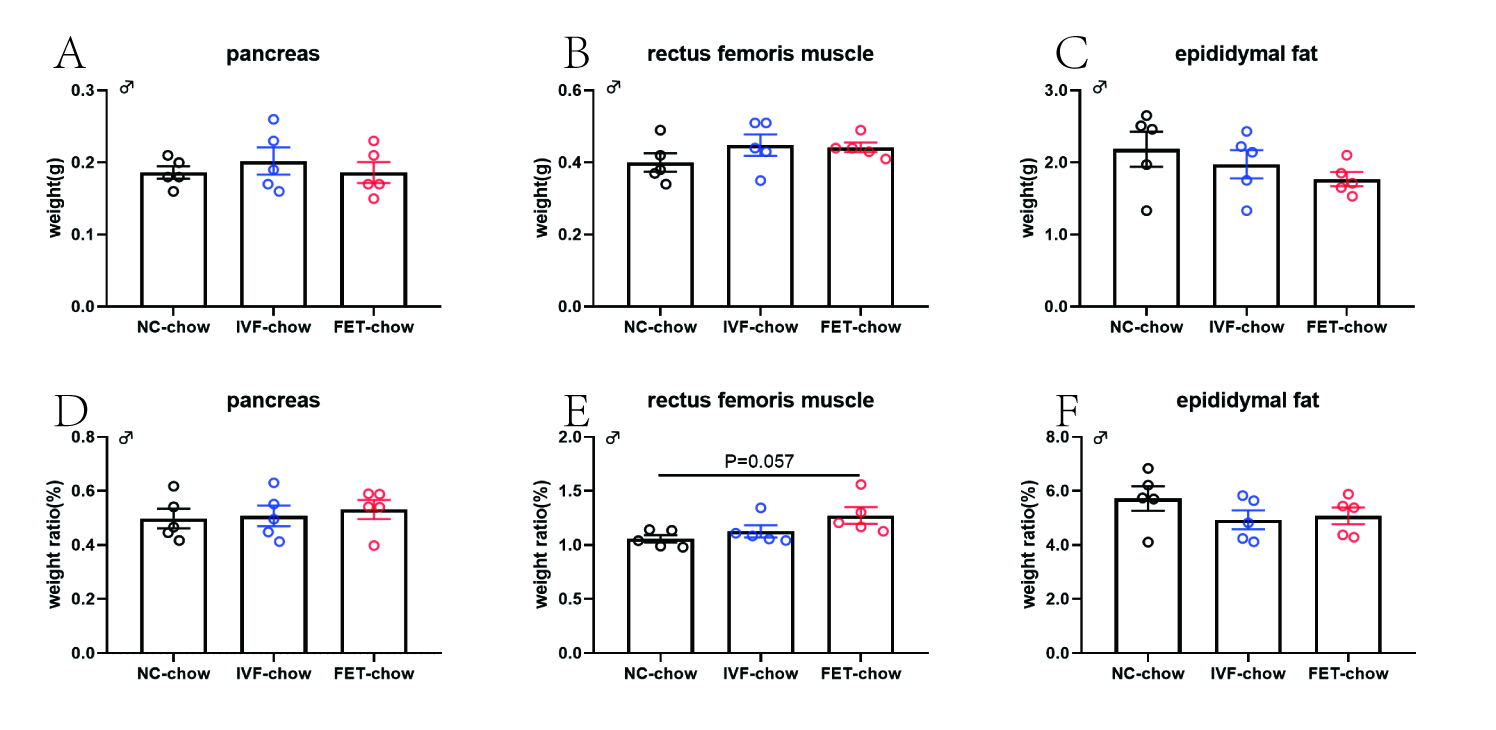

Supplement: Supplementary Figure 2 — Organ weights and ratios in chow-fed male offspring. (A–C) Pancreas, rectus femoris muscle, and epididymal fat weights in chow-fed male offspring at 20 weeks old (n = 5 mice per group). (D–F) Pancreas, rectus femoris muscle, and epididymal fat weight/body weight ratios in chow-fed male offspring at 20 weeks old (n = 5 mice per group). All data were expressed as the mean ± SEM. male offspring at 20 weeks old (n = 5 mice per group). All data were expressed as the mean ± SEM. [file Image_2.TIF]

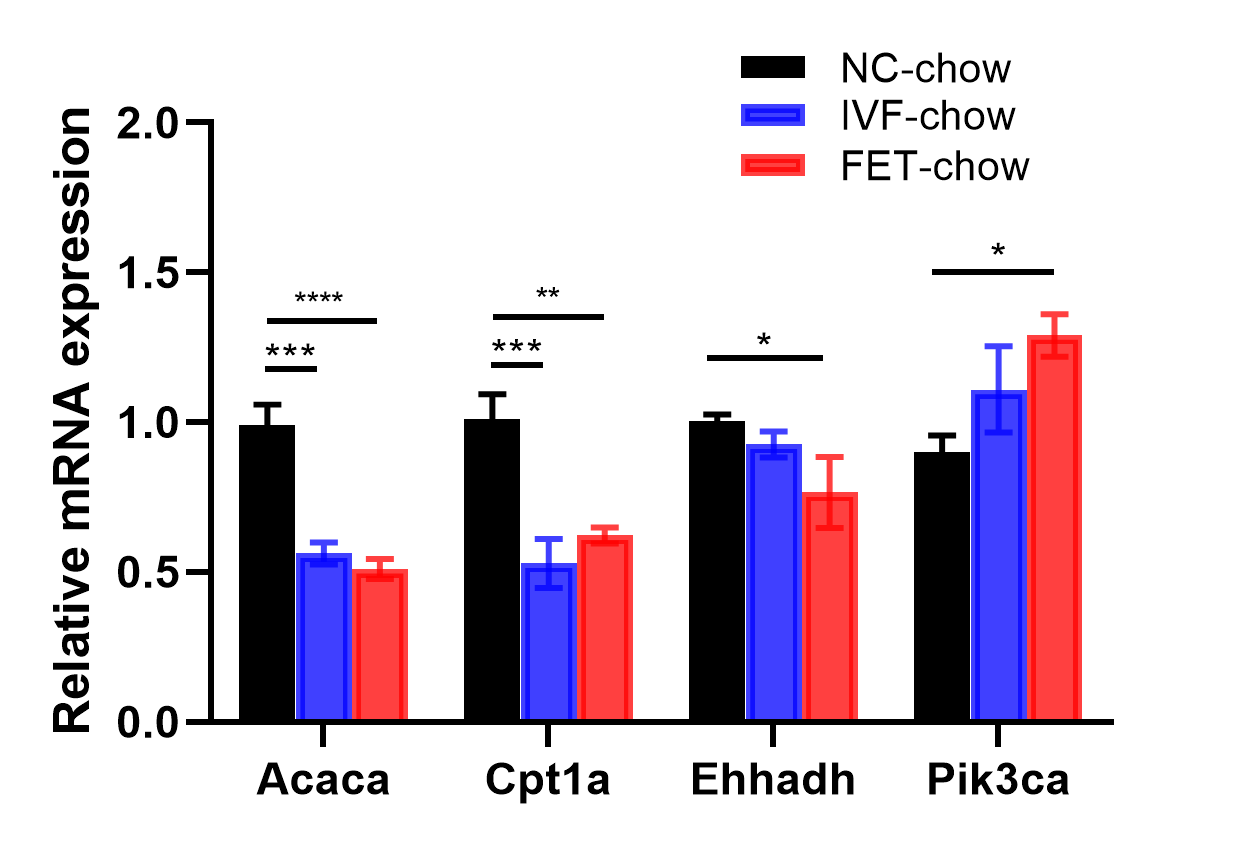

Supplement: Supplementary Figure 3 — The qPCR validation of differential expression genes in sequencing data. Relative mRNA expression levels in the livers of offspring at 20 weeks old (n = 4 mice per group). All data were expressed as the mean ± SEM. [file Image_3.TIF]

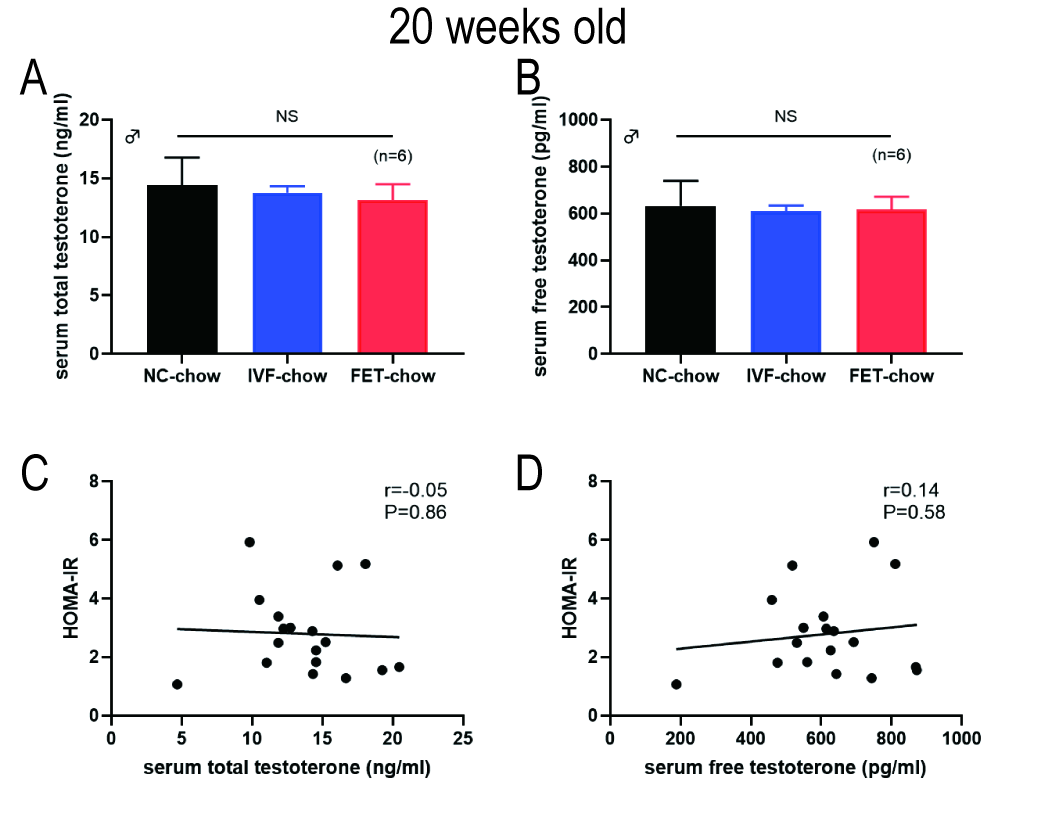

Supplement: Supplementary Figure 4 — The serum testosterone levels in male offspring. (A) Serum total testosterone level in offspring at 20 weeks old (n = 6 mice per group). (B) Serum free testosterone level in offspring at 20 weeks old (n = 6 mice per group). (C) The correlation between serum total testosterone and HOMA-IR in male offspring at 20 weeks old (n = 18). (D) The correlation between serum free testosterone and HOMA-IR in male offspring at 20 weeks old (n = 18). All data were expressed as the mean ± SEM. [file Image_4.TIF]

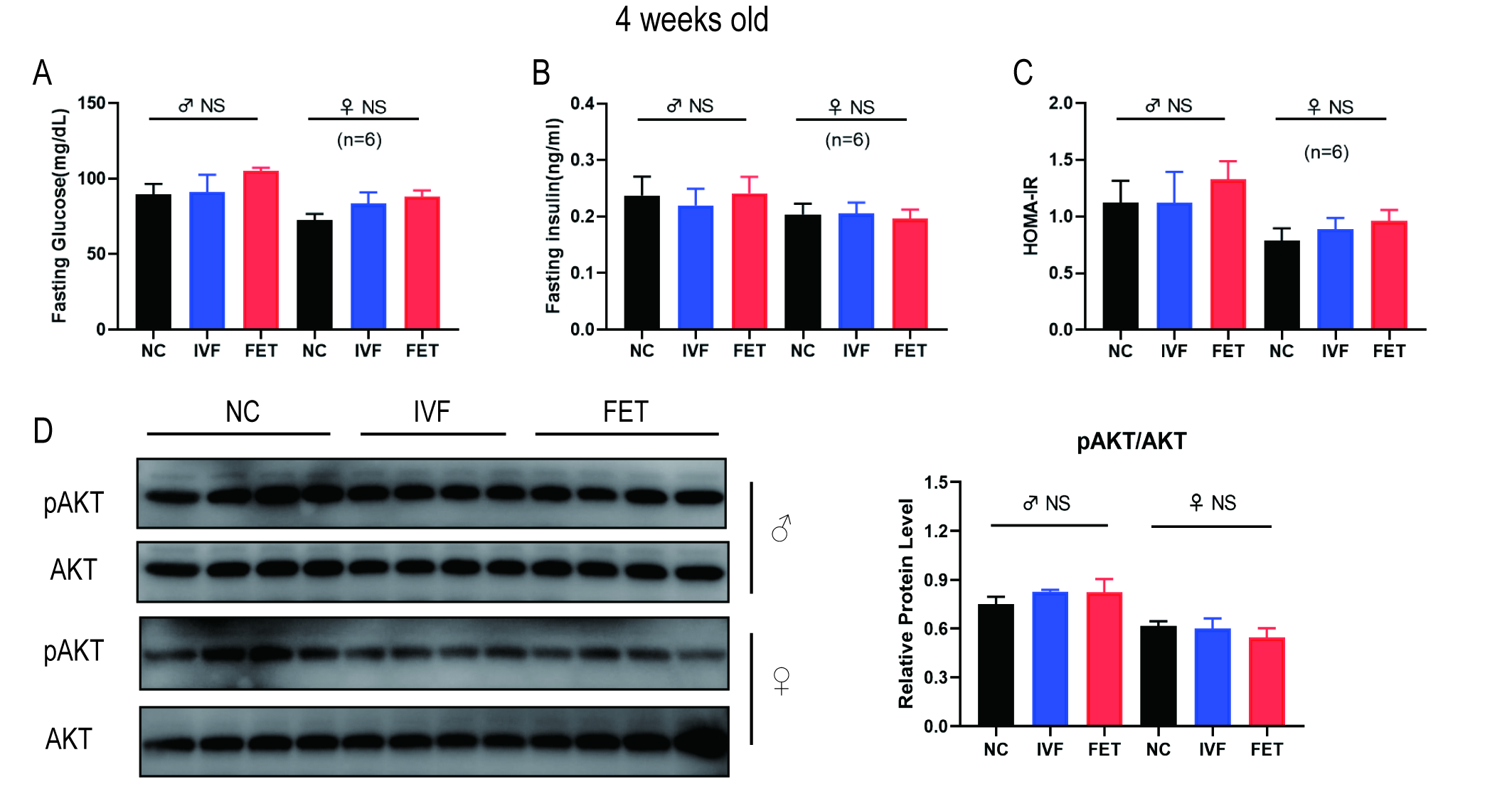

Supplement: Supplementary Figure 5 — The serum fasting glucose, insulin level and relative pAKT/AKT protein expression in 4-week-old offspring. (A) Serum fasting glucose level in offspring at 4 weeks old (n = 6 mice per group). (B) Serum fasting insulin level in offspring at 4 weeks old (n = 6 mice per group). (C) HOMA-IR index in offspring at 4 weeks old (n = 6 mice per group). (D) Relative pAKTn chow-fed male offspring livers at 20 weeks old (n = 4 mice per group). All data were expressed as the mean ± SEM. [file Image_5.TIF]
