# Supplementary material for: Abnormal Glucose Metabolism in Male Mice Offspring Conceived by in vitro Fertilization and Frozen-Thawed Embryo Transfer
Source: Front Cell Dev Biol. 2021 Feb 9;9:637781. doi: 10.3389/fcell.2021.637781 (PMC7900417; doi:10.3389/fcell.2021.637781)

p-IR(Tyr1150/1151)

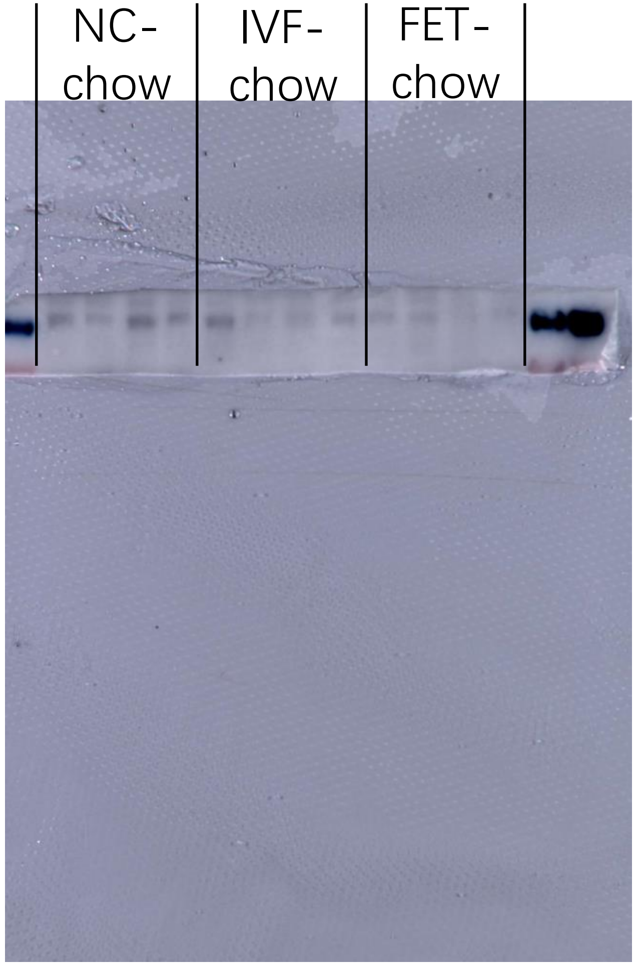

IR

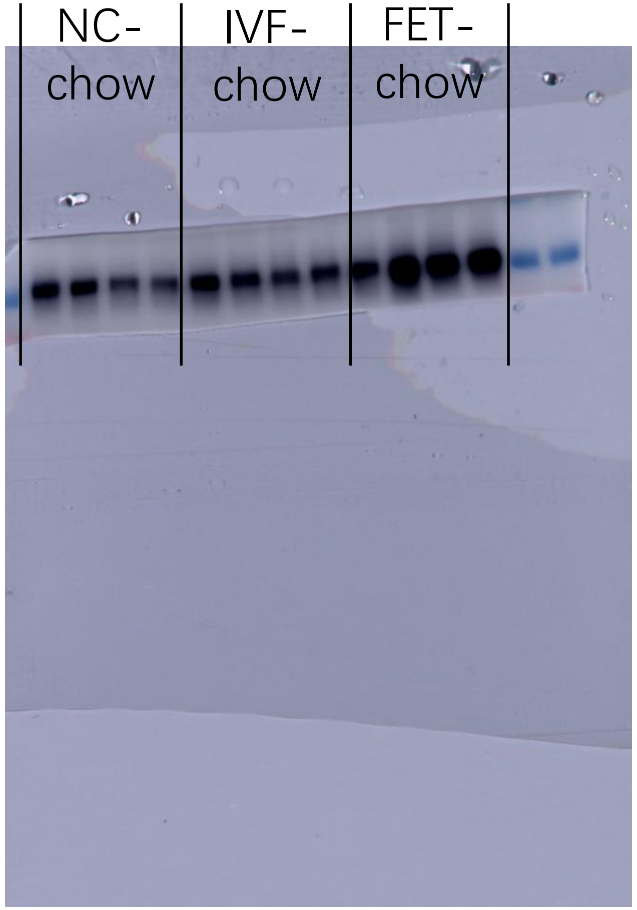

IRS-1

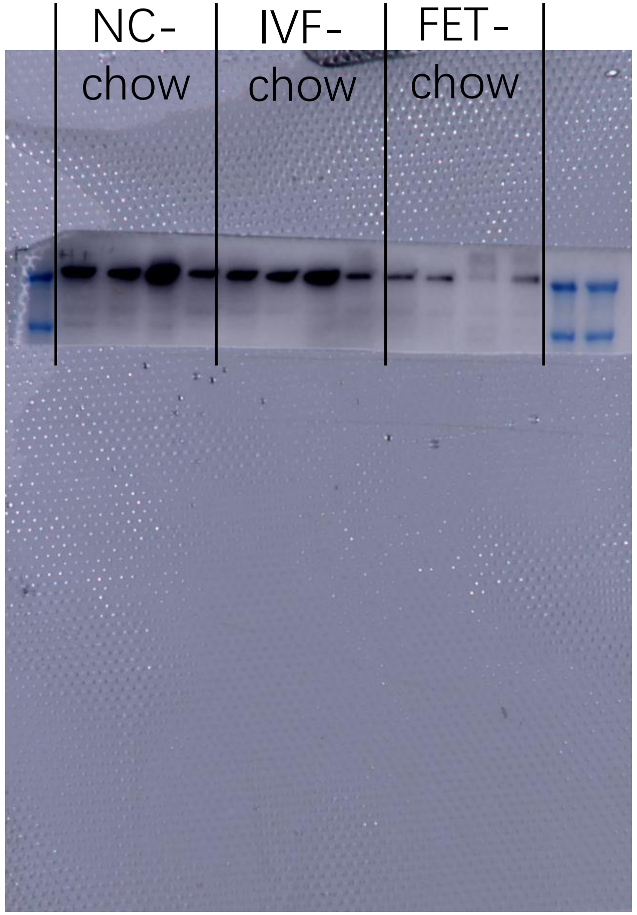

p-AKT(ser473)

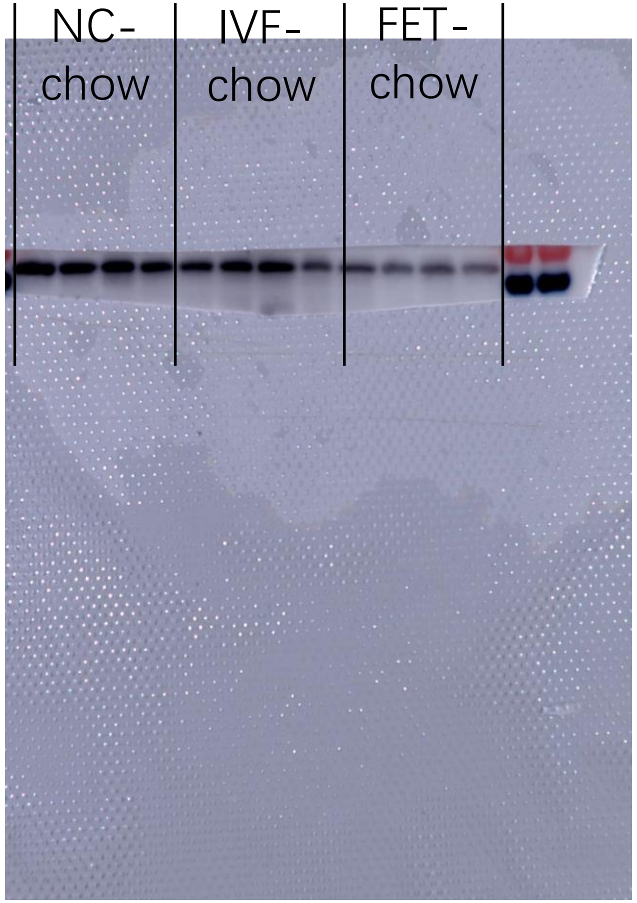

AKT

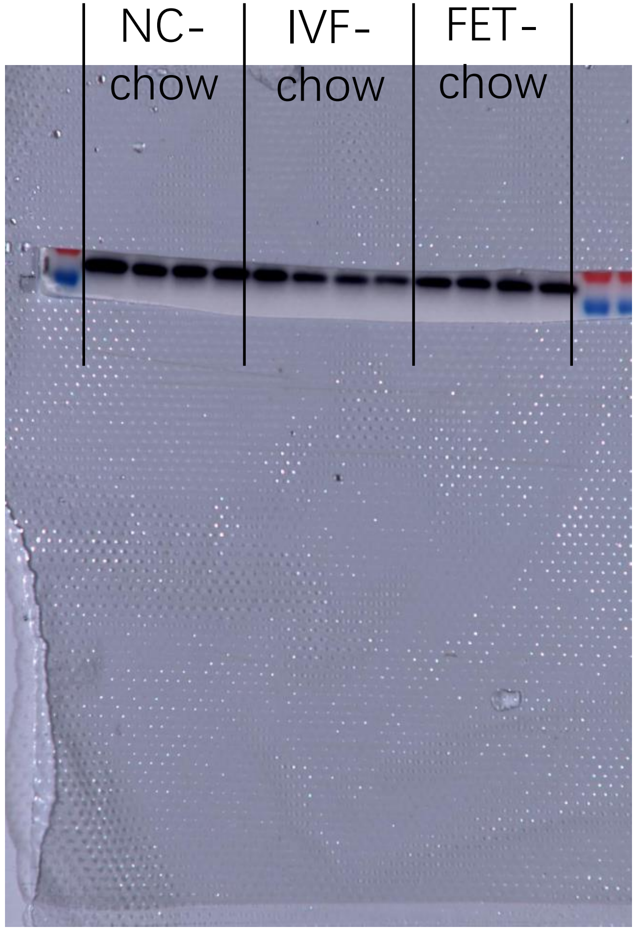

GLUT2

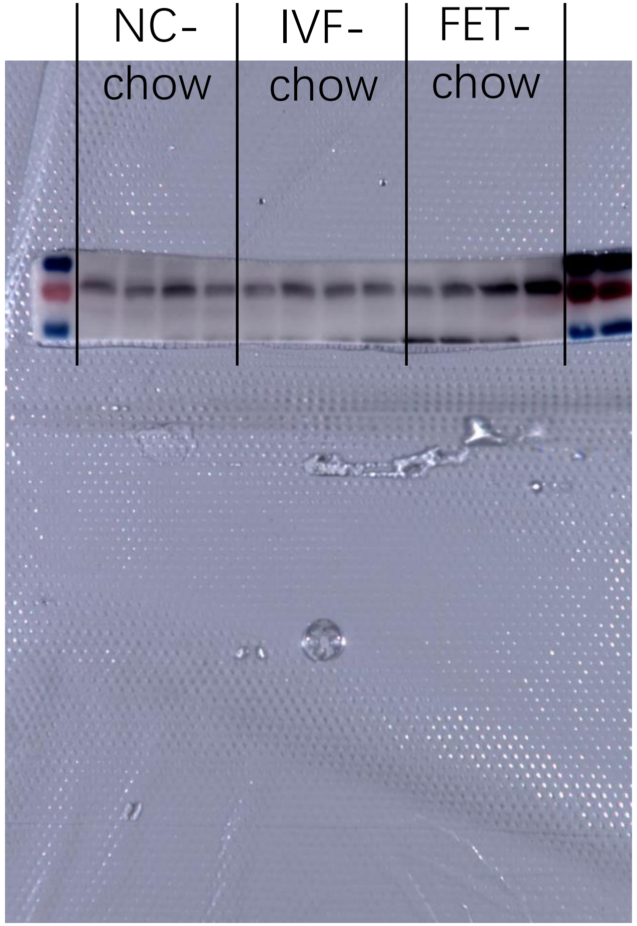

GAPDH(left in Fig. 4H)

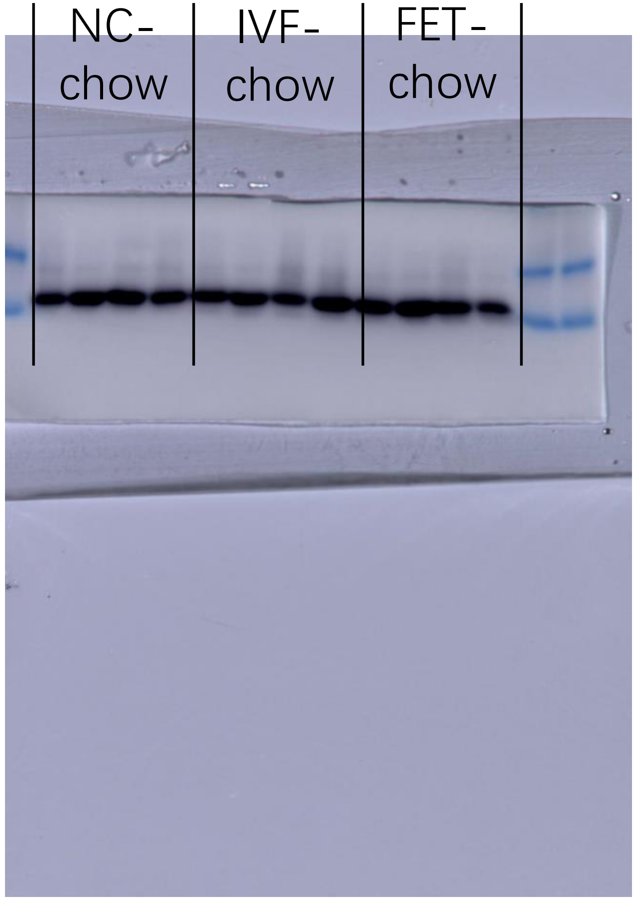

p-foxo1(ser329)

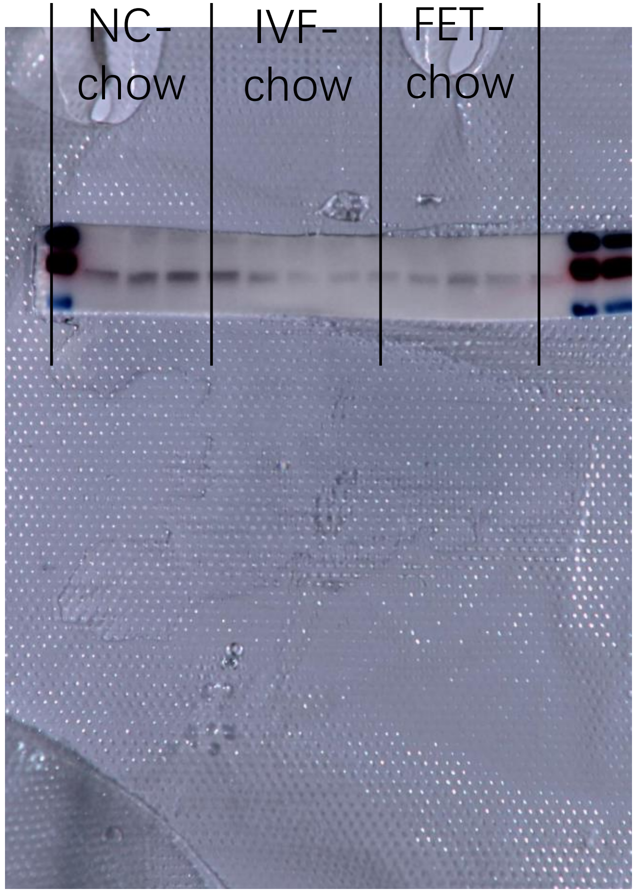

foxo1

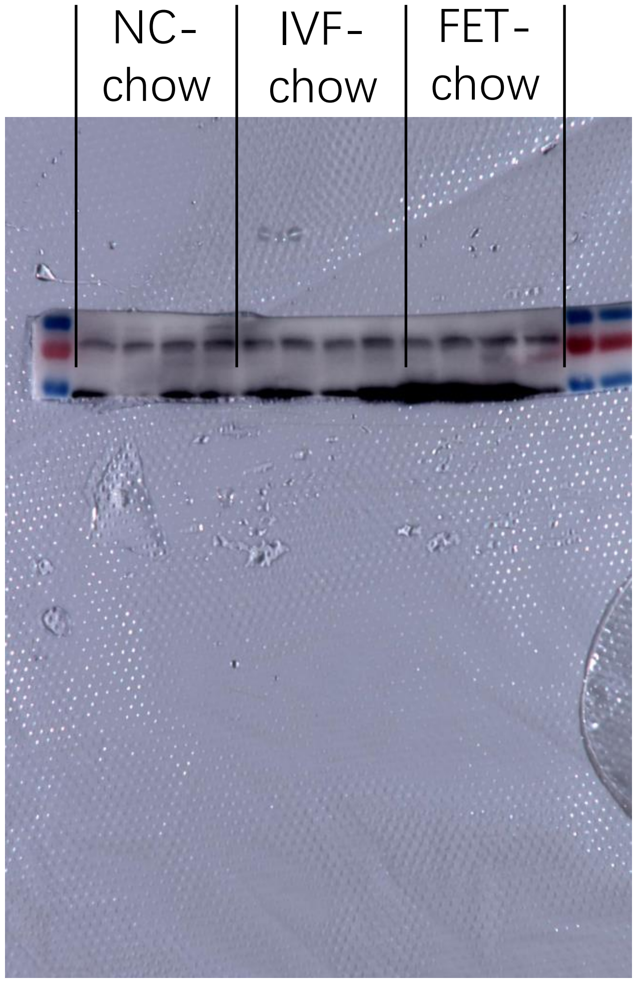

p-GSK3β(Ser9)

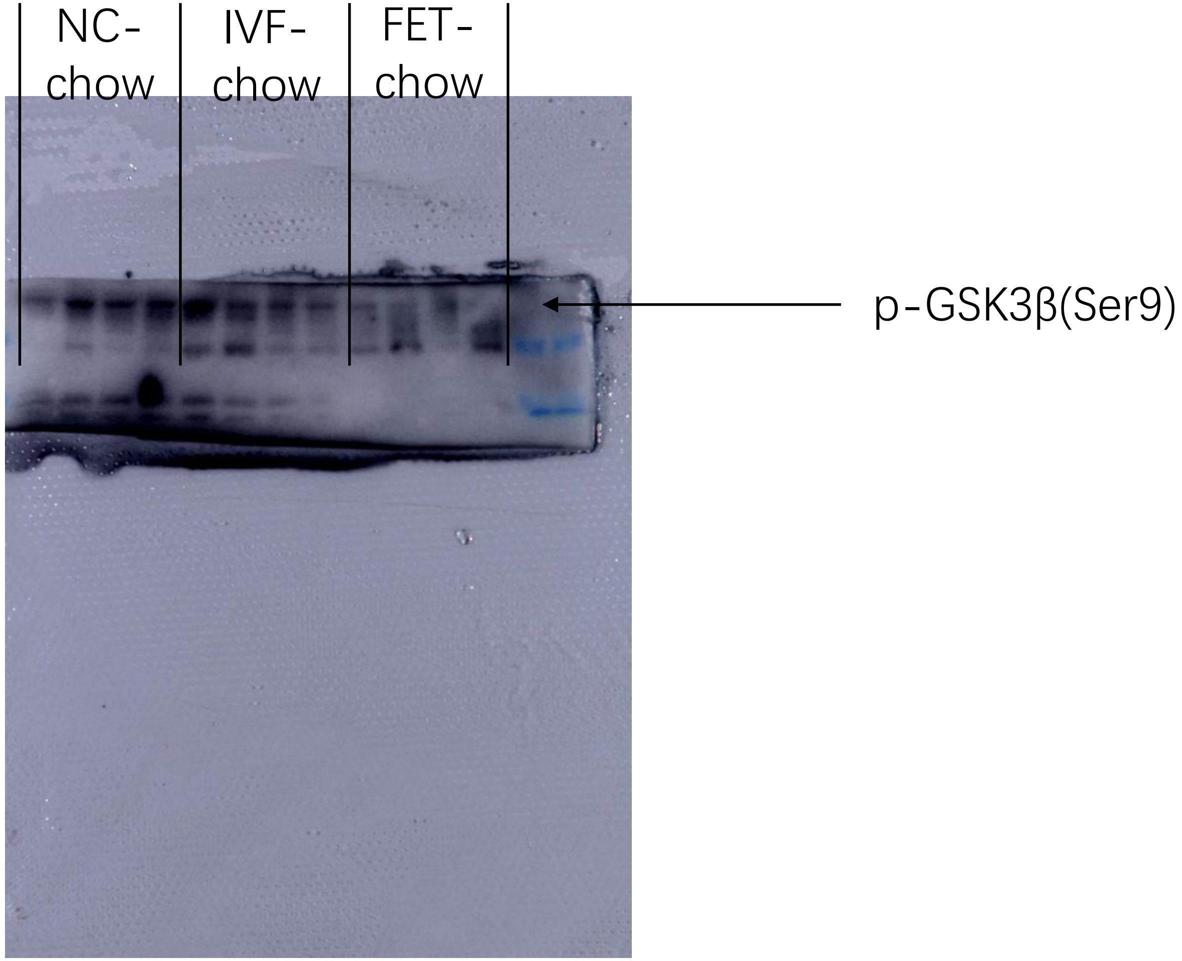

GSK3-β

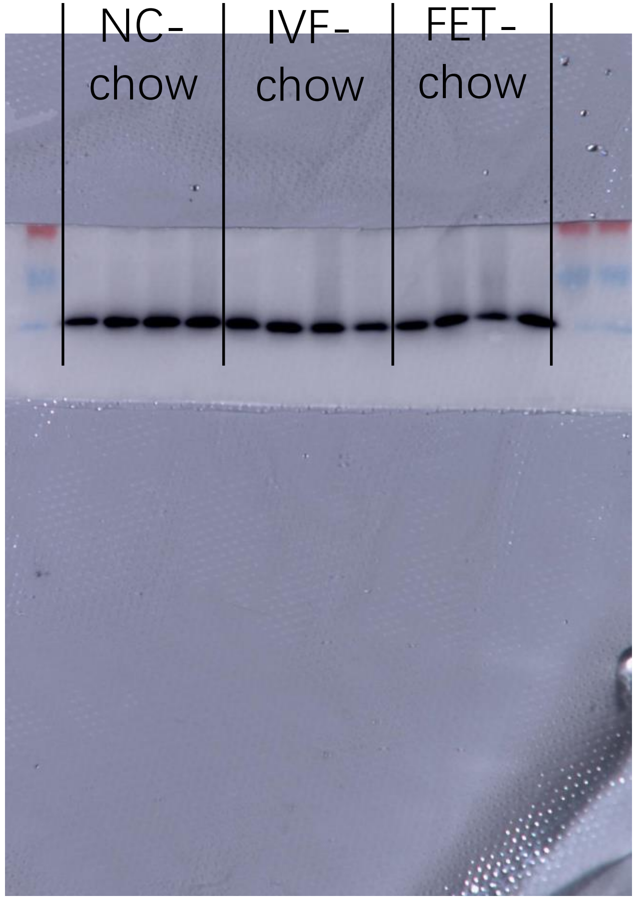

G6PC

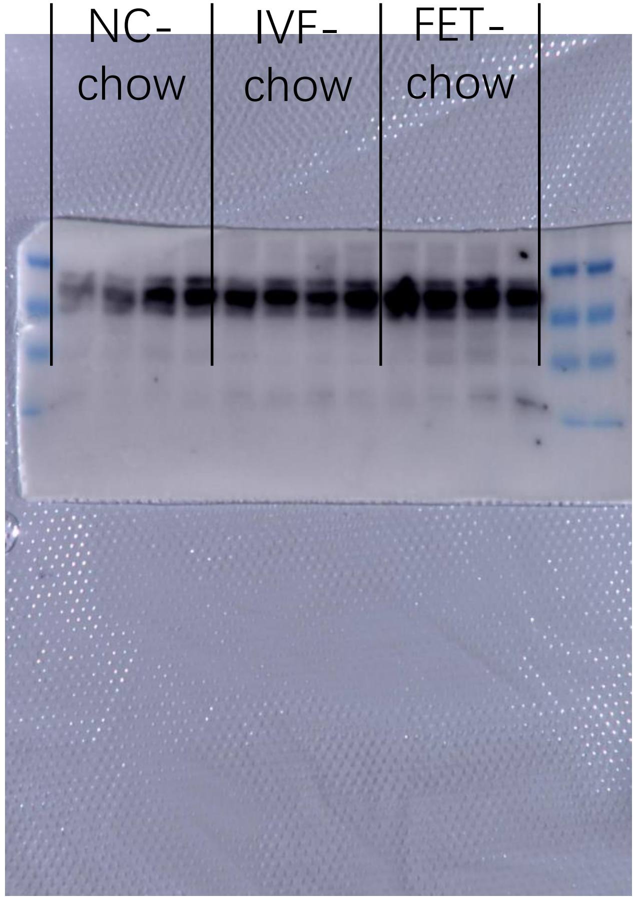

PEPCK

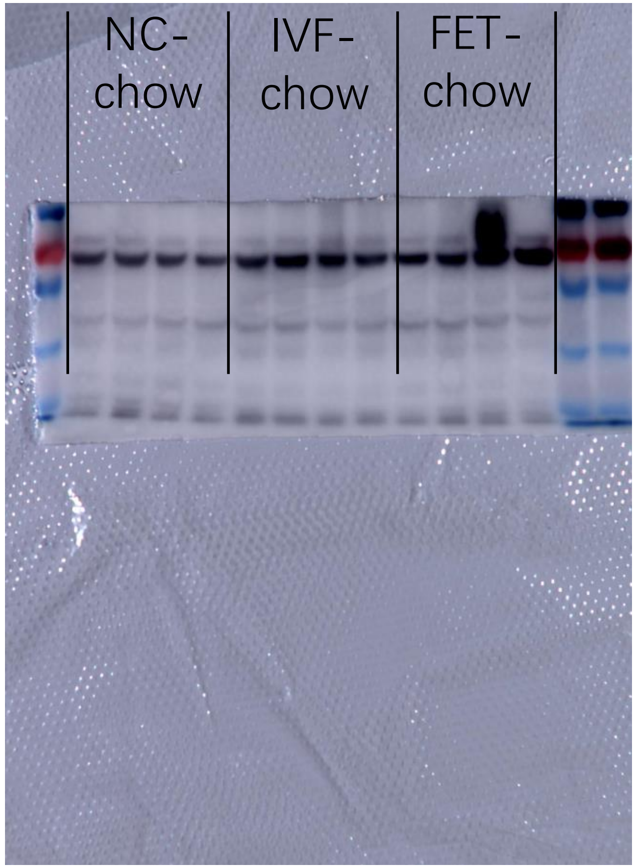

GAPDH(right in Fig. 4H)

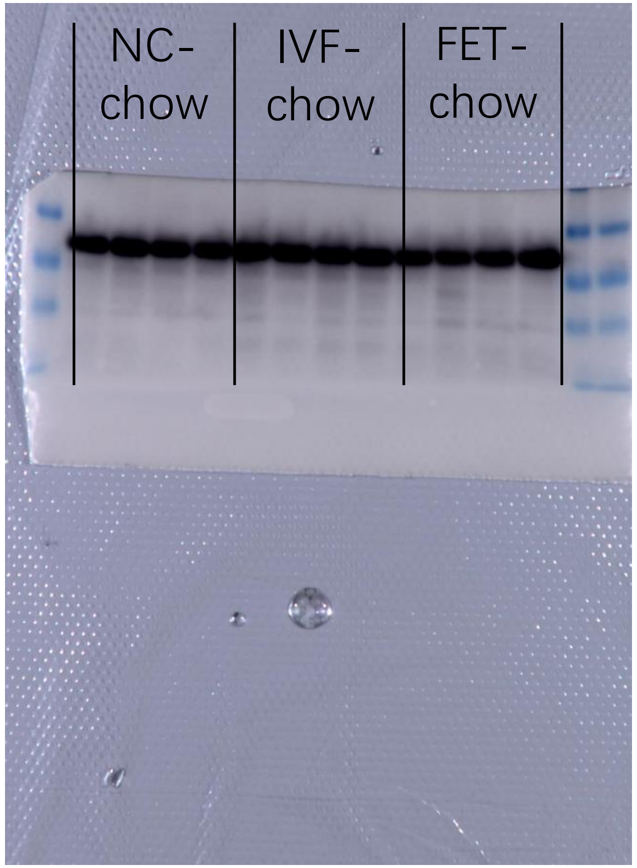

Supplement: Supplementary Material — The entire-unmodified gels related to Figure 4H. [file Image_6.PDF]
