# Supplementary material for: Abnormal Glucose Metabolism in Male Mice Offspring Conceived by in vitro Fertilization and Frozen-Thawed Embryo Transfer
Source: Front Cell Dev Biol. 2021 Feb 9;9:637781. doi: 10.3389/fcell.2021.637781 (PMC7900417; doi:10.3389/fcell.2021.637781)
Supplement: Supplementary file 7 [file Data_Sheet_1.docx]

**Supplementary Table 1**

Primers are used for RT-qPCR. Sequences are printed in the 5’ to 3’direction.

| Transcript | Primers |
| --- | --- |
| GAPDH | F: AGGTCGGTGTGAACGGATTTG  R: GGGGTCGTTGATGGCAACA |
| Acaca | F: CCCAGAGATGTTTCGGCAGTCAC  R: GTCAGGATGTCGGAAGGCAAAGG |
| Cpt1a | F: TGGCATCATCACTGGTGTGTT  R: GTCTAGGGTCCGATTGATCTTTG |
| Ehhadh | F: ACAGCGATACCAGAAGCCAG  R: TGGCAATCCGATAGTGACAGC |
| Pik3ca | F: CACTCGTCACCATCAAACATGA  R: AGGGTTGAAAAAGCCGAAGGT |

**Supplementary Table 2**

Antibodies for Western Blotting

Primary Antibody

| 1^st^ Ab | Company | Cat. No. | Dilution |
| --- | --- | --- | --- |
| GAPDH | Proteintech | 10494-1-AP | 1:5000 |
| p-IR(Tyr1150/1151) | Cell Signaling Technology | 3024 | 1:1000 |
| IR | Cell Signaling Technology | 3025 | 1:1000 |
| IRS-1 | Cell Signaling Technology | 3407 | 1:1000 |
| p-AKT(Ser473) | Cell Signaling Technology | 4060 | 1:1000 |
| AKT | Cell Signaling Technology | 4691 | 1:1000 |
| GLUT2 | Proteintech | 20436-1-AP | 1:1000 |
| p-GSK3-β(Ser9) | Cell Signaling Technology | 5558 | 1:1000 |
| GSK3-β | Cell Signaling Technology | 12456 | 1:1000 |
| p-foxo1(Ser329) | Affinity | AF3416 | 1:1000 |
| Foxo1 | Proteintech | [18592-1-AP](http://www.ptgcn.com/products/FOXO1-Antibody-18592-1-AP.htm) | 1:1000 |
| G6PC | Proteintech | 22169-1-AP | 1:1000 |
| PEPCK | Proteintech | 16754-1-AP | 1:1000 |

Secondary Antibody

| 2^nd^ Ab | Company | Cat. No. | Dilution |
| --- | --- | --- | --- |
| HRP-conjugated Affinipure Goat Anti-Mouse IgG(H+L) | Proteintech | SA00001-1 | 1:10000 |
| HRP-conjugated Affinipure Goat Anti-Rabbit IgG(H+L) | Proteintech | SA00001-2 | 1:10000 |

**Supplementary Table 3** Litter characteristics of offspring.

| group | Pregnancy rate (%) | Implantation rate (%) | Litter(n) | Pups(n) | Litter size(n) | Sex Ratio  (%, M/F) | Survival Rate (%) |
| --- | --- | --- | --- | --- | --- | --- | --- |
| NC | / | / | 15 | 117 | 7.8±0.4 | 1.1(61/56) | 100(117/117) |
| IVF | 73.3(11/15) | 17.3(83/480) | 11 | 83 | 7.5±0.8 | 1.1(43/40) | 100(83/83) |
| FET | 83.3(10/12) | 21.9(84/384) | 10 | 84 | 8.4±0.7 | 1.1(44/40) | 98.8(83/84) |

Data were presented as mean ± SEM; Pregnancy rate: the number of pregnant recipient mice/ the number of all recipient mice; Implantation rate: the number of live pups/ the number of transferred embryos; M/F: male/female. Survival Rate: the number of survival mice after weaning/ the number of all born mice.
